# Effects of different dietary supplements combined with conditioning training on muscle strength, jump performance, sprint speed, and muscle mass in athletes: a systematic review and network meta-analysis

**DOI:** 10.3389/fnut.2025.1636970

**Published:** 2025-07-09

**Authors:** Beiwang Deng, Ruixiang Yan, Tianyuan He, Gesheng Lin, Ting Liu, Wen Chen, Jiaxin He, Duanying Li

**Affiliations:** 1 School of Athletic Training, Guangzhou Sport University, Guangzhou, China; 2 Sports Industry Department, Guangzhou Polytechnic of Sports, Guangzhou, China; 3 School of Physical Education, Guangzhou Sport University, Guangzhou, China; 4 Guangdong Provincial Key Laboratory of Human Sports Performance Science, Guangzhou Sport University, Guangzhou, Guangdong, China

**Keywords:** sport nutrition, ergogenic aids, supplementation, sport performance, sportsman

## Abstract

**Background:**

As dietary supplements have become integral to meeting athletes’ specialised nutritional requirements, research into their effects on performance has intensified. Yet inconsistent findings leave the efficacy of some supplements—most notably branched-chain amino acids and β-hydroxy-β-methylbutyrate (HMB)—open to debate. To clarify which products offer the greatest benefit, we undertook a systematic review and network meta-analysis aimed at identifying the supplements most effective for athletes, including protein, creatine, β-alanine, HMB, vitamin D, caffeine, and others.

**Methods:**

PubMed, Web of Science, the Cochrane Library, Embase and SPORTDiscus were searched from database inception to 15 March 2024 for RCTs evaluating the effects of dietary supplements (including but not limited to protein, creatine, β-alanine, HMB, caffeine, and vitamin D) on athletic performance. Risk of bias was appraised with the revised Cochrane RoB 2 tool. A network meta-analysis was performed in R.

**Results:**

We included 35 randomized controlled trials comprising 991 athletes who completed strength and conditioning training in conjunction with various dietary supplements or a placebo. The risk of bias assessment indicated that 8.57% of studies were at low risk, 88.57% had some concerns, and 2.86% were at high risk of bias. Protein supplementation yielded the greatest improvement in muscular strength (standardized mean difference [SMD] = 0.64, 95% confidence interval [CI] 0.31–0.97; surface under the cumulative ranking curve [SUCRA] = 99.6%; very low-certainty evidence). Both β-alanine (SMD = 0.41, 95% CI 0.10–0.72; SUCRA = 89.0%; moderate-certainty evidence) and creatine (SMD = 0.30, 95% CI 0.07–0.53; SUCRA = 76.06%; moderate-certainty evidence) significantly enhanced jump performance, with β-alanine ranking marginally higher. Creatine also reduced sprint time (SMD = −0.42, 95% CI − 0.68 to −0.16; SUCRA = 94.57%; moderate-certainty evidence). No supplement significantly increased lean body mass.

**Conclusion:**

Protein supplementation appears to be the most effective strategy for increasing muscular strength; β-alanine and creatine both improve jump performance, with β-alanine offering marginally superior effectiveness; and creatine is particularly beneficial for sprint speed. As none of the supplements meaningfully increased muscle mass, practitioners should align supplementation strategies with the targeted performance attribute and training phase to optimise the synergy between nutrition and training and maximise athletic outcomes.

**Systematic review registration:**

PROSPERO, CRD420251048402.

## Graphical abstract

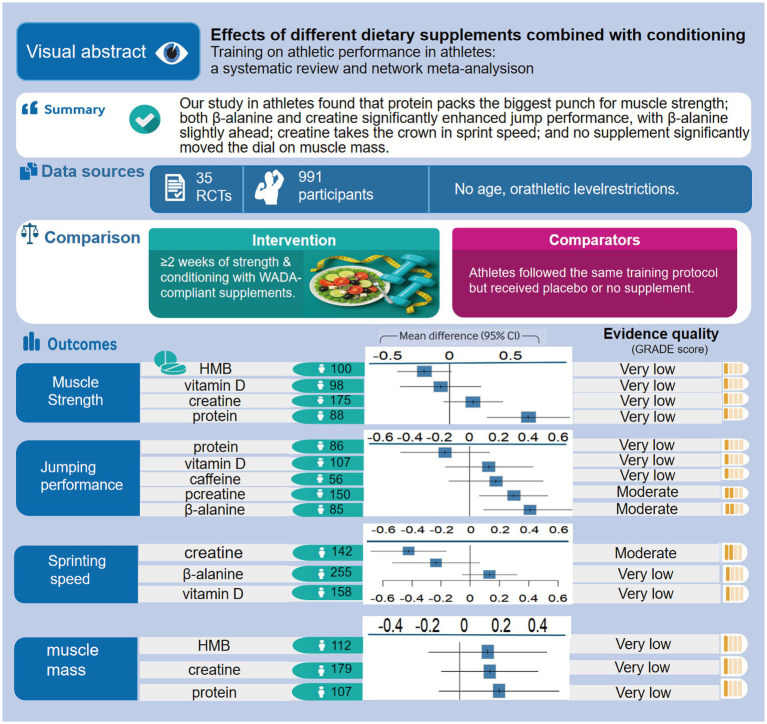

## 1 Introduction

The professionalisation and commercialisation of modern sport, congested competition schedules and high-intensity training loads have made strength and conditioning (S&C) one of the core means of enhancing athletic performance and maintaining competitiveness. Extensive evidence shows that systematic S&C interventions elicit favourable adaptations in maximal strength, power, aerobic and anaerobic endurance, and muscle hypertrophy (1, 2). However, training alone may not sufficiently address the complex physiological demands placed on athletes during periods of intense training and competition. Consequently, the synergistic application of dietary supplements in conjunction with S&C programs has attracted increasing research interest. This combined approach is being investigated for its potential to optimize neuromuscular function, facilitate muscle adaptation, and accelerate post-exercise recovery (3, 4). For instance, protein supplementation has been shown to promote muscle protein synthesis following resistance training, thereby enhancing muscular strength (5). Similarly, ergogenic aids such as creatine and β-alanine have demonstrated promising effects in improving explosive power and short-duration high-intensity performance (6). These findings suggest that an evidence-informed integration of targeted supplementation strategies with systematic S&C training may confer enhanced training adaptations and competitive advantages for athletes.

To meet this demand, nutrition-based strategies—particularly the prudent use of dietary supplements—are increasingly recognised as indispensable for ensuring adequate energy supply and augmenting athletic performance (3, 4). The International Olympic Committee (IOC) consensus statement notes that supplements can enhance physical or cognitive performance, accelerate recovery from strenuous exercise and prevent nutrient deficiencies (7). Insufficient energy intake or an imbalanced macronutrient diet can impair adaptation and recovery and may lead to loss of fat-free mass, immune suppression, reduced bone mineral density, greater injury risk and a higher incidence of over-training syndrome (4, 8). Targeted supplementation has therefore become standard practice in high-performance sport. A meta-analysis of 10,274 athletes reported that 46% of collegiate and 59% of elite competitors use supplements (9), mainly for performance enhancement, faster recovery and health maintenance (8, 10). Certain supplements are even correlated with higher win rates, underscoring their practical value (11).

Despite the widespread use of dietary supplements, the specific effects of different products on training adaptations and athletic performance vary considerably, and systematic evidence comparing the magnitude of these effects remains scarce. Currently, only a limited number of supplements—such as creatine, caffeine, buffering agents (e.g., β-alanine, sodium bicarbonate), and dietary nitrates—have received relatively consistent empirical support, demonstrating significant benefits for strength, explosive power, sprint speed, or training adaptations (12, 13). Creatine increases phosphocreatine stores and boosts high-intensity performance, particularly amplifying strength and power gains during resistance training (14). Caffeine raises neuromuscular activation and alertness, enhancing jump and sprint performance (15). Buffering agents delay the accumulation of acidic metabolites, improving high-intensity intermittent exercise capacity (16). Protein supplements (e.g., whey) supply essential amino acids that promote muscle repair and hypertrophy, strengthening gains in both force and muscle mass (17).

Determining the efficacy of dietary supplements and tailoring their use to individual goals and needs is pivotal to the success of nutritional interventions (18). Nevertheless, the performance effects of many widely marketed “trending” supplements remain inadequately defined. For instance, although branched-chain amino acids may attenuate muscle soreness, multiple studies have not demonstrated superiority over placebo for strength or performance enhancement (19). β-Hydroxy-β-methylbutyrate (HMB) is touted for its anti-catabolic properties; however, its additional benefits in trained populations are limited (20). Pre-workout blends exhibit modest acute ergogenic effects, yet robust evidence for sustained improvements is lacking (21). This divergence between supplement popularity and empirical support impedes practitioners’ ability to select products that optimally augment strength, power, speed, or muscle hypertrophy.

Network meta-analysis (NMA) provides methodological advantages for addressing this issue. By integrating direct and indirect evidence, NMA systematically compares and ranks multiple interventions within a single analytic framework (22). Such an approach allows a comprehensive assessment of the synergy between S&C and supplementation strategies and offers practitioners robust evidence. Accordingly, we performed the first NMA to systematically evaluate randomized controlled trials that combined S&C with diverse supplementation protocols (focusing on commonly utilized supplements such as protein, creatine, β-alanine, HMB, caffeine, and vitamin D, among others), comparing their effects on maximal strength, jump performance, sprint speed, and lean body mass, and ranking the overall efficacy of each regimen. By synthesising the available evidence, we sought to identify the most effective dietary supplements for enhancing athletic performance and to provide practitioners—including sports dietitians, coaches, and exercise scientists—with recommendations for tailoring nutritional strategies to individual needs.

## 2 Methods

### 2.1 Study registration

This review was conducted in accordance with the Preferred Reporting Items for Systematic Reviews and Network Meta-Analyses (PRISMA-NMA) statement (23) and was prospectively registered in PROSPERO (ID: CRD420251048402). Given the complexity of comparing multiple interventions, adherence to the PRISMA-NMA guidelines ensured methodological rigour.

### 2.2 Search strategy

We searched PubMed, Web of Science, Embase, SPORTDiscus and the Cochrane Central Register of Controlled Trials. Clinical trial registries were also screened for unpublished data, and the reference lists of all included studies were examined to identify additional citations. No restrictions were imposed on region, year or publication language. These databases were chosen for their comprehensive coverage of sports nutrition and sports science literature. The search spanned from database inception to 15 March 2025. The complete search strategy is provided in Appendix 1.

### 2.3 Eligibility criteria

The eligibility criteria were defined according to the PICOS framework. Population: only systematically trained athletes of any sex or age were considered. Interventions: trials had to combine at least two weeks of structured strength and conditioning (24) with one or more dietary supplements that contained no substance listed on the World Anti-Doping Agency Prohibited List; supplements could be provided singly or in combination. Comparators: control groups were required to follow the identical training protocol—matching frequency, intensity, and supervision—while receiving a placebo or no supplement. Outcomes: studies had to report at least one post-intervention performance measure related to maximal strength, jump performance, sprint speed, or muscle mass (Table 1). Study design: eligible studies were restricted to randomised controlled trials, irrespective of blinding status (open-label, single-blind, or double-blind); both parallel-group and crossover designs were accepted, provided crossover trials incorporated an adequate washout period and, if carry-over effects were anticipated, analysed data from the first treatment period only.

**Table 1:** Performance measures.

Outcome indicators	Exercise test
Muscular strength	Upper body strength or lower body strength tests.
Jump Performance	Countermovent jump, vertical jump, squat jump, and other jump ability tests.
Sprint Speed	Short sprint tests such as 10-meter, 20-meter and 30-meter sprints.
Muscle mass	Lean body mass by DXA, multi-frequency BIA, or skinfold-derived fat-free mass.

Studies were excluded if they (i) involved animals; (ii) enrolled injured or clinical populations; (iii) investigated supplementation with carbohydrate or caffeine alone, or formulations in which carbohydrate or caffeine was combined with other supplements; (iv) failed to specify the exact dose or timing of the supplement; (v) were not published in English; (vi) lacked full-text availability; (vii) were not peer-reviewed (e.g., conference abstracts, theses, grey literature); or (viii) were non-original articles such as reviews, opinion pieces, commentaries, case reports, or editorials.

### 2.4 Study selection and data extraction

All records were imported into Zotero 7. Titles and abstracts were screened to identify potentially eligible studies; full texts of these studies were then reviewed against the inclusion criteria. A standardised extraction form captured: title, first author, publication year, study design, country, intervention characteristics, intervention duration, sample size, sex, age, sport discipline, performance tests and outcome variables. Two reviewers (BW and RX) independently screened and extracted data, cross-checking results on completion. Discrepancies were resolved by consensus or, when outcome definitions were unclear, with the assistance of a third reviewer (GS). Inter-rater reliability for study selection was calculated using Cohen’s kappa statistic (Almost Perfect, Cohen’s kappa = 0.83).

### 2.5 Measures of treatment effect

In this meta-analysis, intervention effects were expressed as the change in mean difference (MD) and standard deviation (SD). When an original study did not report the SD directly, we derived it from the standard error (SE), 95% confidence interval (CI), *p*-value or *t*-statistic, following published guidance (25). For trials that lacked the SD of the pre-to-post change, we calculated it with the equation:


$$SD_{\text{change}}=\sqrt{SD_{\text{baseline}}^{2}+SD_{\text{post}}^{2}-2r\;SD_{\text{baseline}}\;SD_{\text{post}}}$$

Assuming a correlation coefficient (*r*) of 0.5 between baseline and follow-up measurements (26). This moderate value, commonly adopted in the literature, balances potential variability between repeated measures and thus supports the robustness and reliability of the pooled estimates.

### 2.6 Quality assessment of evidence

We evaluated each trial with the Cochrane Risk of Bias tool for randomized controlled trials (RoB 2.0), covering random sequence generation, allocation concealment, blinding, missing outcome data and selective outcome reporting (27). A study was classified as “high overall risk of bias” if at least one domain was rated as “high risk” (score = 1). A study was classified as having “some concerns” if no domain was rated “high risk” but at least one domain was rated as “some concerns” (score = 2). A study was classified as “low overall risk of bias” if all domains were rated as “low risk” (score = 3). Two reviewers (BW and RX) conducted the assessments independently and resolved disagreements through discussion or, when necessary, consultation with a third reviewer (GS). Inter-rater reliability for the RoB 2.0 domain-level assessments was also calculated using Cohen’s kappa statistic (Substantial, Cohen’s Kappa = 0.72).

Certainty of evidence for every network estimate was graded using the Confidence in Network Meta-Analysis (CINeMA) framework (28). This approach evaluates six domains: (i) within-study bias (based on RoB 2.0 assessments of contributing studies), (ii) reporting bias (assessed via funnel plots and Egger’s test where appropriate), (iii) indirectness (considering PICO alignment and transitivity), (iv) imprecision (determined by the 95%CI width), (v) heterogeneity (statistical and clinical), and (vi) incoherence (consistency between direct and indirect evidence, assessed via node-splitting) (28, 29). Detailed criteria for evaluating each CINeMA domain, specific conditions leading to rating concerns, and downgrading rules are provided in Appendix S1: Detailed CINeMA Assessment Protocol. Two reviewers (BW and RX) independently assessed certainty, with discrepancies resolved by discussion or a third reviewer (GS).

### 2.7 Statistical analysis

A frequentist network meta-analysis was performed in R 4.3.3 using the netmeta package and a graph-theoretical approach. Effect sizes were obtained via weighted least-squares regression solved with the Moore–Penrose generalised inverse, and a random-effects model was applied to account for between-study heterogeneity (30, 31). When outcomes shared an identical scale, results were pooled as MD; otherwise, SMD with 95%CI were calculated for comparability.

Global and local heterogeneity were assessed with the generalised Cochran Q statistic. Potential sources of heterogeneity, such as differences in participant characteristics, training protocols, and supplement dosages, were considered qualitatively. Inconsistency between direct and indirect evidence was examined with node-splitting; a *p* < 0.05 indicated significant inconsistency (32).

The network structure was illustrated with a network plot in which nodes represent interventions and edges denote direct comparisons, allowing for a visual assessment of the network geometry (connectivity and distribution of direct evidence). To explore potential bias related to the network structure and describe the evidence base, key characteristics of included studies (participant demographics, intervention details, outcome measures; see Section 2.4 and Appendix 2) were systematically collected and reviewed to assess transitivity. Relative effects were summarised in forest plots and league tables. Intervention rankings were derived from the surface under the cumulative ranking curve (SUCRA) and visualised with a rank heat map (33, 34). Publication bias was further checked with funnel plots and Egger’s regression test. The magnitude of the SMD was interpreted using Cohen’s (68) conventional criteria, where an SMD of approximately 0.2 was considered a small effect, 0.5 a moderate effect, and 0.8 a large effect (35).

## 3 Results

### 3.1 Search results

**Figure 1:**
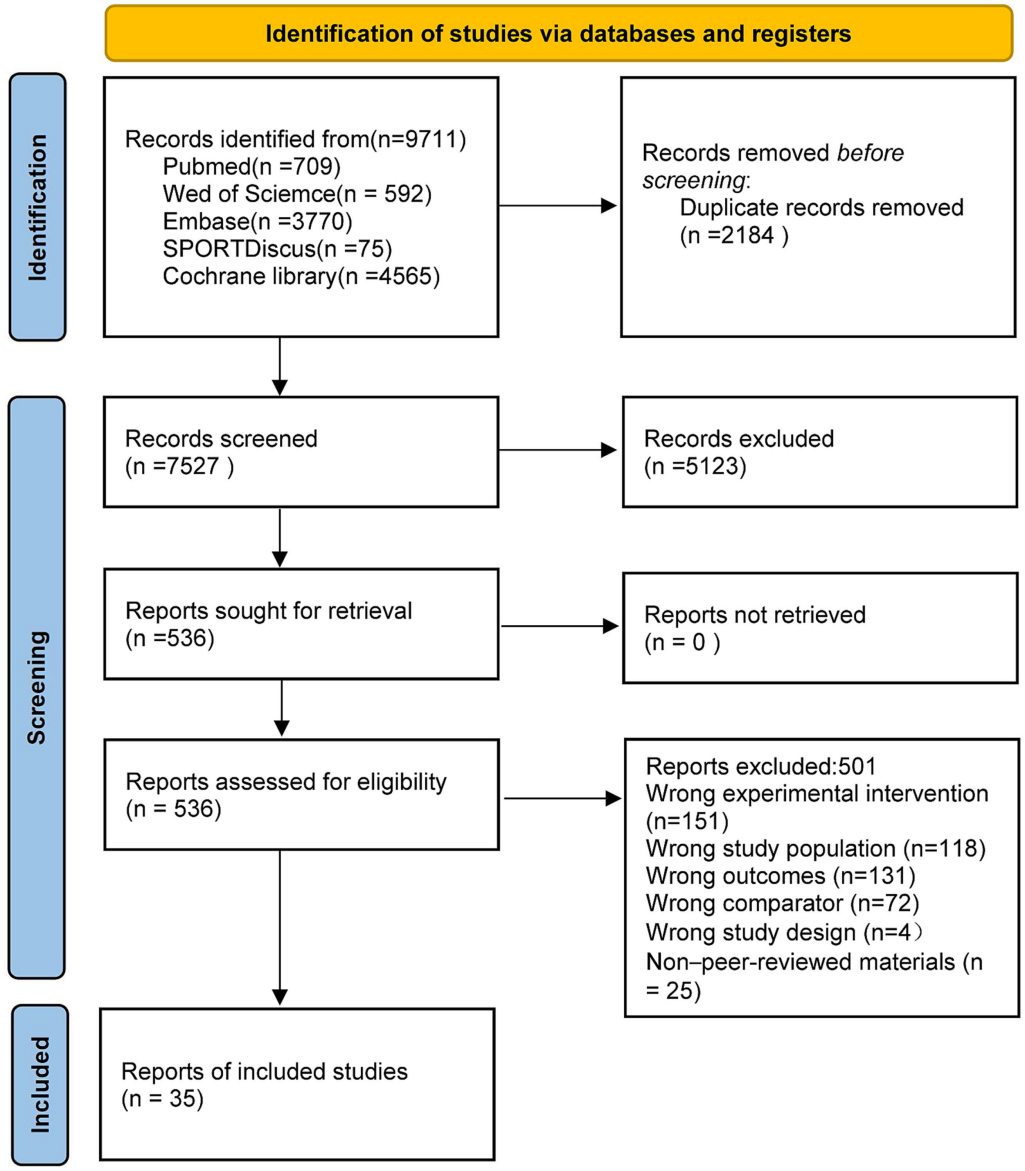
Flowchart of screening process.

Database searches yielded 9,711 records, of which 35 studies met the inclusion criteria and were entered into the network meta-analysis. The selection process is illustrated in Figure 1, which shows the numbers of records screened and excluded at each stage. After removing 2,184 duplicates from different databases, titles and abstracts of 9,711 records were screened, and 536 full-text articles were assessed for eligibility. Ultimately, 35 published randomised controlled trials fulfilled all criteria and were included in the quantitative synthesis. The complete screening and selection flow is presented in Figure 1.

The 35 included RCTs were published between 1999 and 2025, with a combined total of 991 systematically trained athletes, comprising 831 males (83.9%) and 160 females (16.1%). Studies involved athletes from diverse sports [e.g., team sports like soccer (36), baseball athletes (37)]. Mean ages typically ranged from ~17 years (38) to ~30 years (39), mostly young adults (18–25 years). Intervention durations were 2–12 weeks. Common supplements included creatine, β-alanine, protein, and vitamin D. Detailed characteristics are in Appendix 2 (Table S2.1). Inter-rater agreement for study eligibility assessment based on full-text review was substantial (Almost Perfect, Cohen’s kappa = 0.85).

### 3.2 Risk of Bias

**Figure 2:**
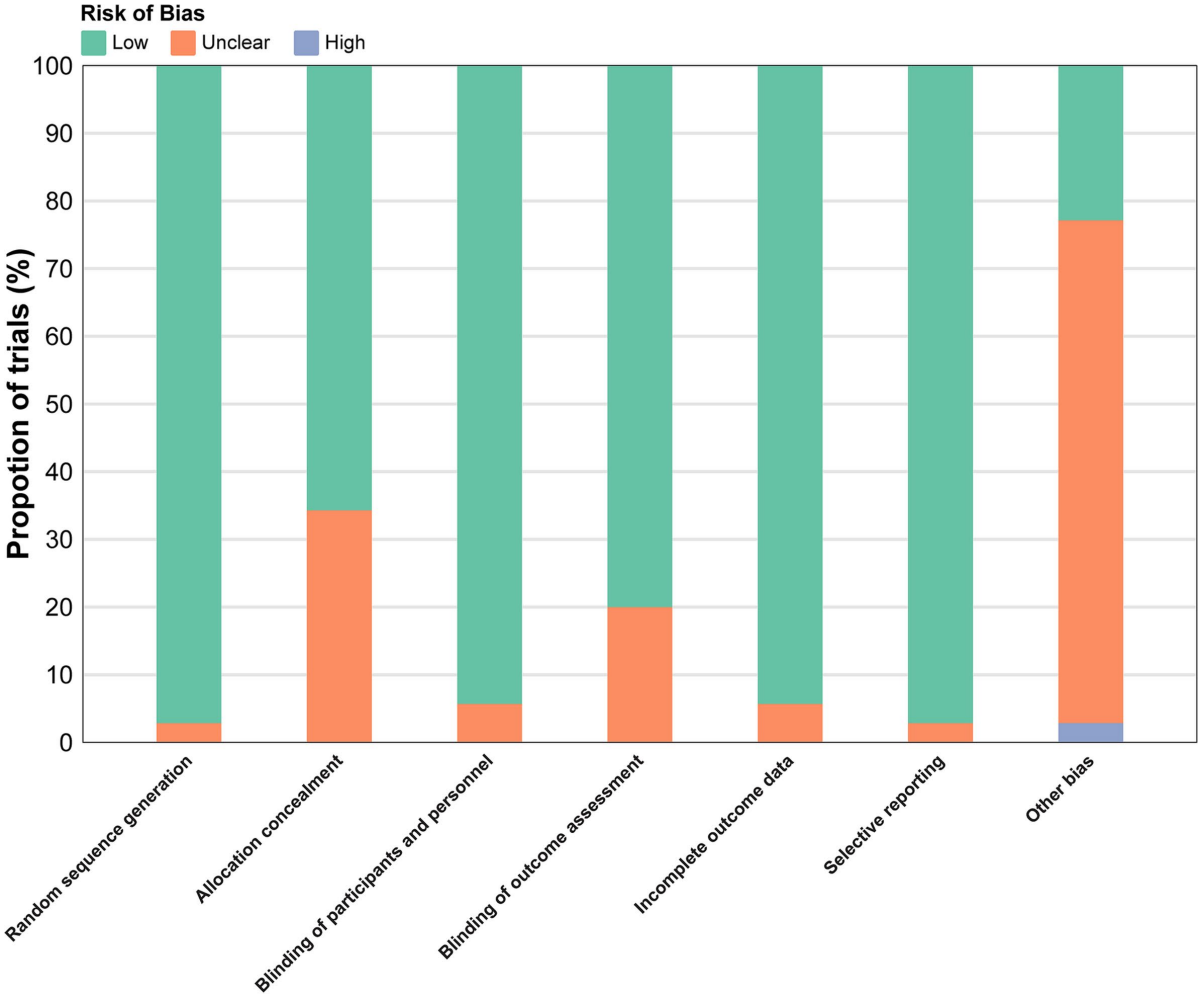
Overall risk of bias presented as percentage of each risk of bias item across all included.

Overall, 3 studies (8.57%) were low risk of bias, 31 (88.57%) had some concerns, and 1 (2.86%) was high risk (Figure 2), often due to deviations from intended interventions or missing data. Detailed assessments are in Appendix 3 (Figure S2).

### 3.3 Certainty of evidence

Using the CINeMA framework (CINeMA framework; Methods Section 2.6; Appendix S1: Detailed CINeMA Assessment Protocol), certainty for pairwise comparisons ranged from very low to moderate (Appendix 8). All networks satisfied the transitivity assumption, supporting the validity of indirect estimates.

### 3.4 Network geometry, consistency, and heterogeneity

**Figure 3:**
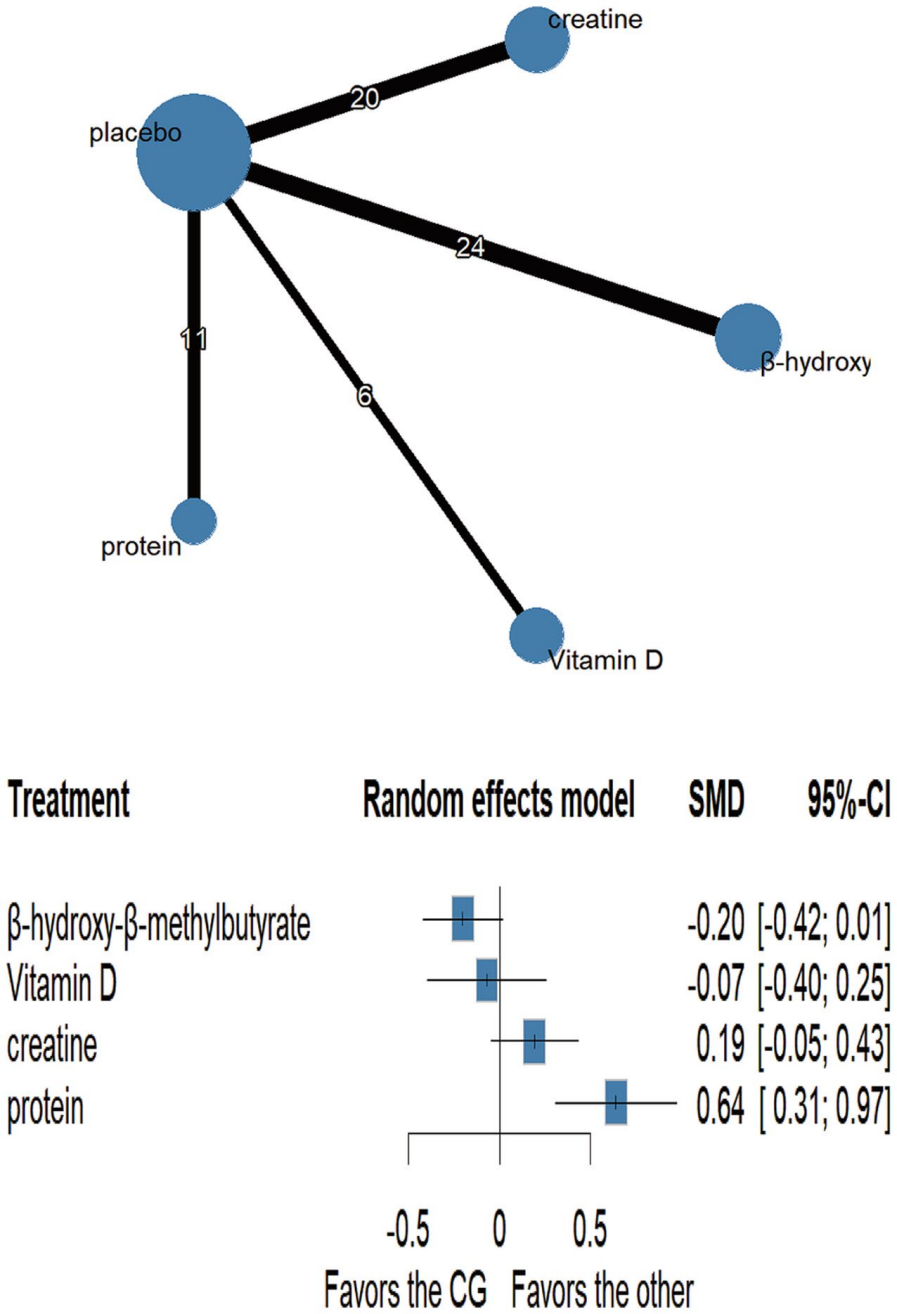
Network plot and forest plot of interventions for muscle strength.

**Figure 4:**
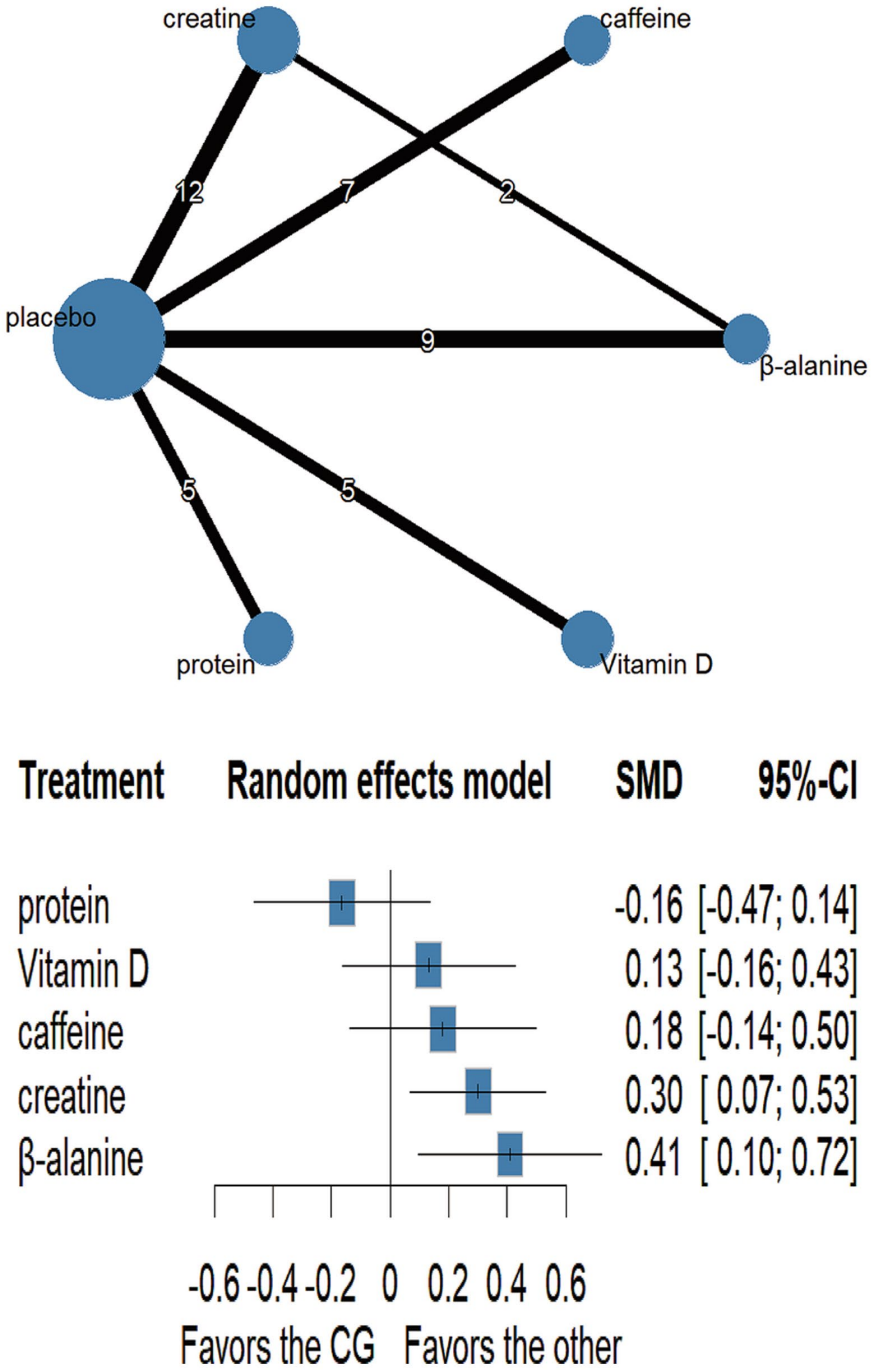
Network plot and forest plot of interventions for jumping performance.

**Figure 5:**
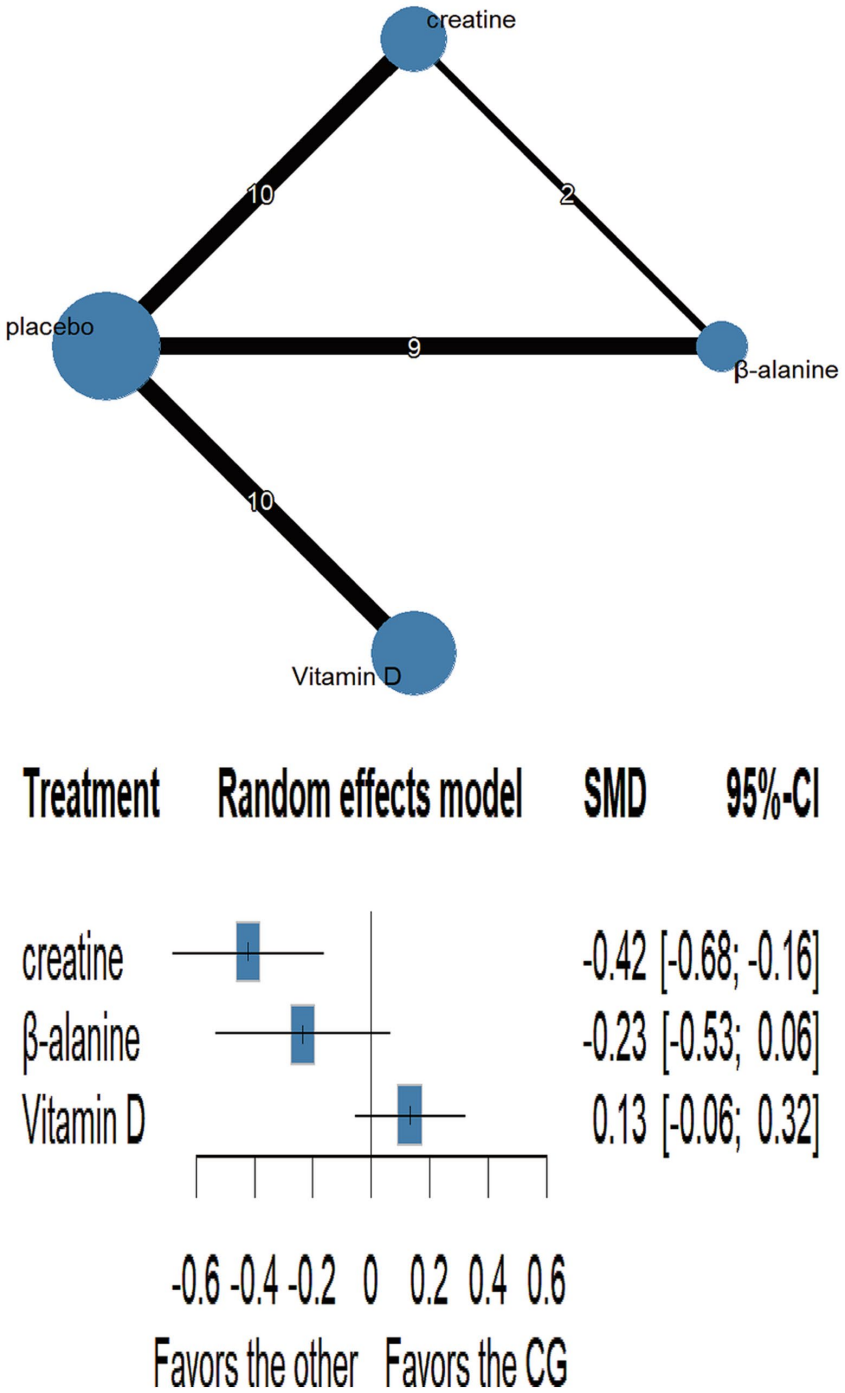
Network plot and forest plot of interventions for sprinting speed.

**Figure 6:**
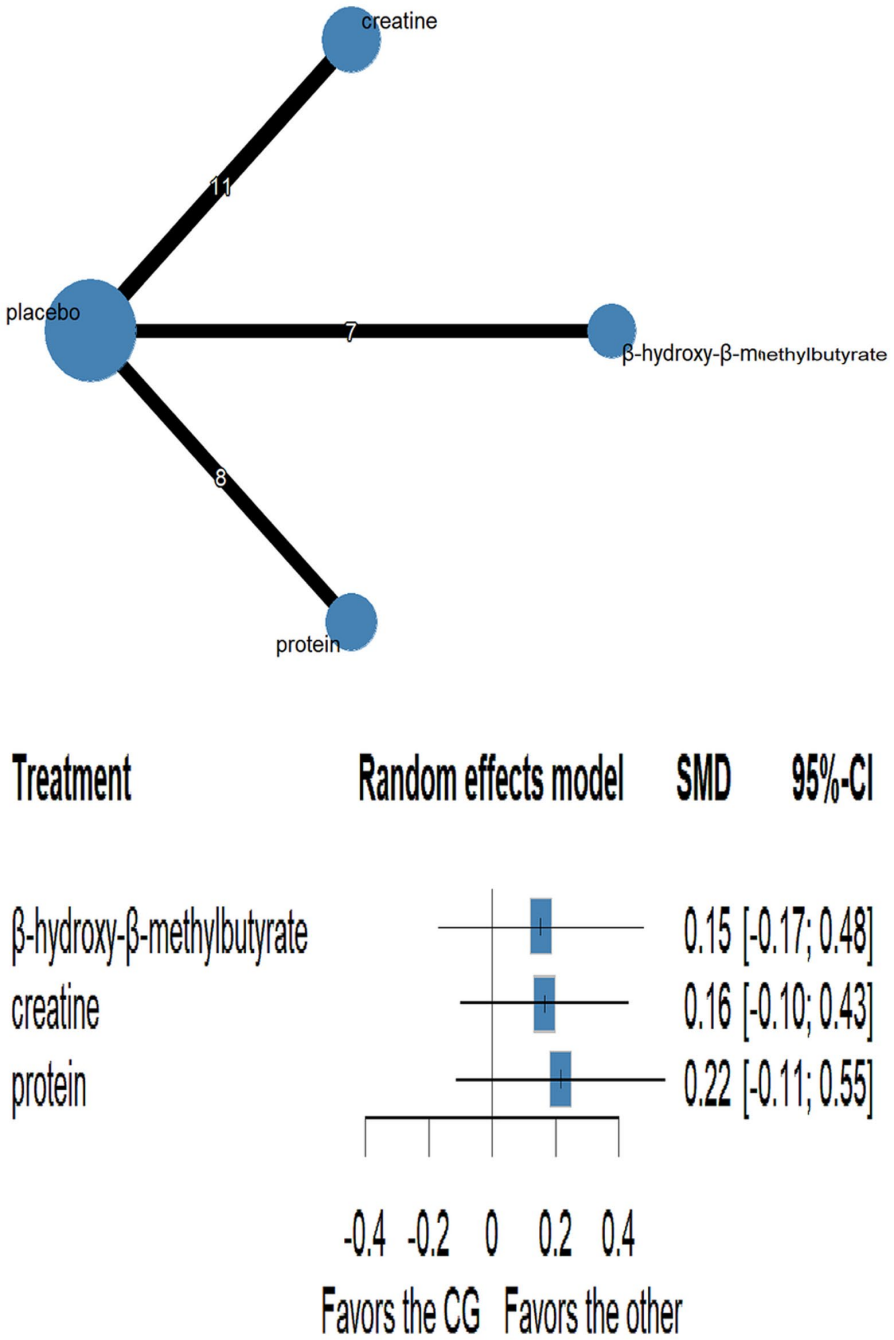
Network plot and forest plot of interventions for muscle mass.

Network plots are shown in Figures 3–6 (Appendix 5, Figures S5.1–S5.4). Node-splitting analyses detected no significant inconsistency for jumping or sprinting performance (*p* > 0.05; Appendix 4, Tables S4.3 and S4.5), whereas formal tests were infeasible for muscular strength and muscle mass because their networks lacked closed loops; results for these outcomes should therefore be interpreted with caution. Statistical heterogeneity was moderate for muscular strength (τ^2^ = 0.0947, I^2^ = 35.5%; Appendix 4, Table S4.1) and negligible for jumping performance, sprinting performance, and muscle mass (τ^2^ = 0, I^2^ = 0%; Appendix 4, Tables S4.2, S4.4, S4.6). Comparison-adjusted funnel plots and Egger’s regression tests revealed no evidence of publication bias (Appendix 9, Figures S9.1–S9.4).

### 3.5 Meta-analysis

#### 3.5.1 Muscular strength

**Figure 7:**
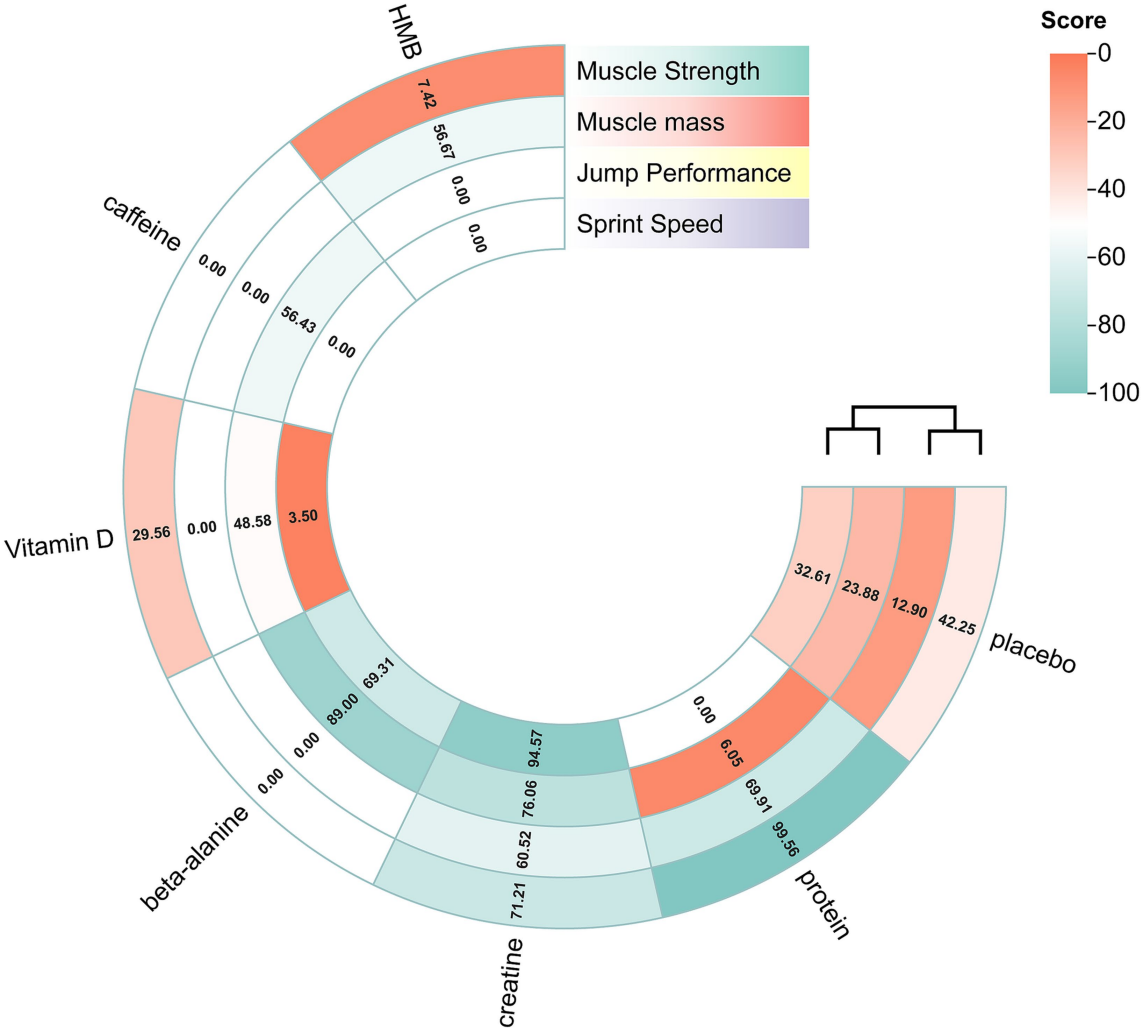
Rank-heat plot obtained from treatment-level network meta-analysis. The rank heat plot presents a summary of *P* scores (range 0–100) for each intervention across outcomes, where darker shades of green represent more benefit and darker shades of red represent less benefit. Beta-alanine = β-alanine; HMB = β-hydroxy-β-methylbutyrate.

Fifteen randomised trials involving 442 athletes compared the effects of four dietary supplements on muscular strength. Very-low-certainty evidence indicated that protein supplementation produced a significant improvement over control (SMD = 0.64, 95%CI 0.31–0.97; SUCRA = 99.56%). By contrast, HMB, vitamin D and creatine showed no clear benefits: HMB (SMD = −0.20, 95% CI − 0.42 to 0.01; SUCRA = 7.42%), vitamin D (SMD = −0.07, 95% CI − 0.40 to 0.25; SUCRA = 29.56%) and creatine (SMD = 0.19, 95% CI − 0.05 to 0.43; SUCRA = 71.21%). Detailed network estimates are presented in Figures 3, 7 and Table 2.

**Table 2:** Ranking of the effects of different nutritional interventions on muscular strength.

Creatine				
−0.43 (−0.82; −0.04)	Protein			
0.33 (0.02; 0.64)	0.76 (0.38; 1.13)	HMB		
0.26 (−0.12; 0.63)	0.69 (0.25; 1.12)	−0.07 (−0.43; 0.30)	Vitamin D	
0.19 (−0.04; 0.42)	0.61 (0.30; 0.93)	−0.14 (−0.35; 0.07)	−0.07 (−0.37; 0.23)	Placebo

#### 3.5.2 Jump performance

Fourteen trials involving 460 athletes evaluated five dietary supplements for their effects on jump performance. Moderate-certainty evidence indicated that β-alanine produced the greatest improvement (SMD = 0.41, 95% CI 0.10–0.72; SUCRA = 89.0%) and ranked as the most effective intervention. Creatine also significantly enhanced jump performance (SMD = 0.30, 95% CI 0.07–0.53; SUCRA = 76.06%), placing it among the top-ranked strategies. By contrast, protein, vitamin D and caffeine showed no clear benefit: protein (SMD = −0.16, 95% CI − 0.47 – 0.14; SUCRA = 6.05%), vitamin D (SMD = 0.13, 95% CI − 0.16 – 0.43; SUCRA = 48.58%) and caffeine (SMD = 0.18, 95% CI − 0.14 – 0.50; SUCRA = 56.43%). Detailed estimates are presented in Figures 4, 7 and Table 3.

**Table 3:** Ranking of the effects of different nutritional interventions on jump performance.

Creatine					
0.46 (0.08; 0.84)	Protein				
−0.11 (−0.48; 0.26)	−0.57 (−1.01; −0.14)	β-alanine			
0.12 (−0.27; 0.51)	−0.34 (−0.78; 0.09)	0.23 (−0.22; 0.68)	Caffeine		
0.17 (−0.21; 0.54)	−0.30 (−0.72; 0.13)	0.28 (−0.15; 0.71)	0.05 (−0.39; 0.48)	Vitamin D	
0.30 (0.07; 0.53)	−0.16 (−0.47; 0.14)	0.41 (0.10; 0.72)	0.18 (−0.14; 0.50)	0.13 (−0.16; 0.43)	Placebo

#### 3.5.3 Sprint speed

Ten randomised trials comprising 373 athletes assessed three dietary supplements for their influence on sprint speed. Moderate-certainty evidence showed that creatine produced a significant improvement over control (SMD = −0.42, 95% CI − 0.68 to −0.16; SUCRA = 94.57%), ranking it as the most effective intervention. By contrast, β-alanine (SMD = −0.23, 95% CI − 0.53 to 0.06; SUCRA = 69.31%) and vitamin D (SMD = 0.13, 95% CI − 0.06 to 0.32; SUCRA = 3.5%) did not significantly affect sprint performance. Full estimates are presented in Figures 5, 7 and Table 4.

**Table 4:** Ranking of the effects of different nutritional interventions on sprint speed.

Creatine			
−0.19 (−0.56; 0.19)	β-alanine		
−0.55 (−0.87; −0.24)	−0.37 (−0.72; −0.01)	Vitamin D	
−0.42 (−0.68; −0.16)	−0.23 (−0.53; 0.06)	0.13 (−0.06; 0.32)	Placebo

#### 3.5.4 Muscle mass

Thirteen randomised trials encompassing 402 athletes compared the effects of three dietary supplements on muscle mass. None of the interventions conferred a significant benefit over control: HMB (SMD = 0.15, 95% CI − 0.17 to 0.48; SUCRA = 56.67%), creatine (SMD = 0.16, 95% CI − 0.10 to 0.43; SUCRA = 60.52%) and protein (SMD = 0.22, 95% CI − 0.11 to 0.55; SUCRA = 69.91%). Full network estimates are shown in Figures 6, 7 and Table 5.

**Table 5:** Ranking of the effects of different nutritional interventions on muscle mass.

Creatine			
−0.05 (−0.47; 0.37)	Protein		
0.01 (−0.41; 0.43)	0.06 (−0.40; 0.52)	HMB	
0.16 (−0.10; 0.43)	0.22 (−0.11; 0.55)	0.15 (−0.17; 0.48)	Placebo

## 4 Discussion

### 4.1 Summary of the main findings

This network meta-analysis systematically evaluated the effectiveness of commonly used dietary supplements, when combined with physical conditioning, on muscular strength, jump performance, sprint speed and muscle mass in athletes. Very-low-certainty evidence indicates that protein supplementation significantly enhances muscular strength. Moderate-certainty evidence shows that both β-alanine and creatine markedly improve vertical-jump performance, while creatine also significantly increases sprint speed. None of the supplement strategies produced a statistically significant benefit for muscle mass.

### 4.2 Muscular strength

Dietary protein is the primary substrate for muscle protein synthesis, and substantial empirical evidence supports its efficacy in enhancing muscular strength. Our NMA revealed a moderate effect size (SMD = 0.64) for protein supplementation on strength, ranking it as the most effective intervention. This aligns with protein’s established roles in activating anabolic signaling pathways such as mTOR, supplying essential amino acids during the post-exercise anabolic window, and promoting muscle repair and adaptation (40). Further supporting the role of protein, a comprehensive meta-analysis by Morton et al. (41) involving 49 randomised controlled trials found that protein supplementation, when combined with structured resistance exercise training, significantly increased maximal strength in healthy adults. While Morton and colleagues emphasized that RET itself is the primary driver of strength gains, their findings also indicated a statistically significant, albeit smaller (a small additional benefit, approximately 9% greater increase), from protein supplementation (41). Our NMA, which included studies combining various forms of S&C with supplementation, similarly identified protein as a leading strategy for strength enhancement.

However, under the CINeMA framework, the certainty of evidence was rated as “very low” due to limited indirect comparisons and network inconsistency assessments. While baseline protein intake and energy balance were often inadequately controlled, these were less influential than the absence of indirect evidence. Additionally, findings on protein’s ergogenic effect are not uniform. In some trials involving well-trained individuals with sufficient daily protein intake, supplementation did not lead to further strength improvements (42). Schoenfeld et al. (43) further suggested that when daily protein intake is adequate, the timing of ingestion has little impact on chronic strength outcomes. Another consideration is energy balance: the strength benefit of supplementation is likely amplified when baseline protein intake is inadequate or when participants are in a negative energy state—conditions often underreported in primary studies. These methodological issues likely contributed to the very low certainty rating.

In contrast, we found no significant strength-enhancing effects for creatine (SMD = 0.19), HMB (SMD = −0.20), or vitamin D (SMD = −0.07), each with very low-certainty evidence. Although HMB, a leucine metabolite, is thought to exert anti-catabolic effects by supporting myocellular repair and reducing oxidative stress (38), our findings and recent reviews (39) suggest negligible strength gains in trained athletes. This may be due to HMB’s benefits being limited to untrained or catabolic populations (44). Formulation differences (calcium salt vs. free acid) and dosing variability (1.5–3 g/day) also contribute to outcome heterogeneity. Vitamin D has received attention for its role in muscle function via skeletal muscle vitamin D receptors (VDRs), which regulate calcium balance and mitochondrial activity (45). Yet, its lack of efficacy in our findings may reflect sufficient baseline vitamin D levels in most participants, with benefits typically restricted to deficient individuals (46).

Creatine remains one of the most widely used and researched strength-promoting supplements, primarily by elevating intramuscular phosphocreatine (PCr) stores and thereby accelerating adenosine triphosphate (ATP) resynthesis during brief, high-intensity efforts (14, 47). The absence of a statistically significant overall effect of creatine on maximal strength in our NMA, despite a point estimate suggesting a small positive effect (SMD = 0.19), may stem from several factors. Firstly, the training stimulus itself is crucial. Some studies may have employed S&C programs with insufficient volume, intensity, or progression (“sub-optimal training loads”) to fully elicit or detect creatine’s ergogenic potential on maximal strength. Creatine tends to be more effective when combined with high-intensity resistance training programs that repeatedly challenge the phosphagen system, allowing for greater training volume or intensity to be performed over time (14, 47). Moreover, in highly trained athletes, creatine’s ergogenic advantages often manifest more clearly in dynamic, power–speed actions (e.g., sprinting or jumping), where our NMA did find small to moderate significant effects, rather than in isolated, slower velocity maximal strength tests (14, 48).

### 4.3 Jump performance

Our analysis, based on ‘moderate’ certainty evidence, shows that both β-alanine (SMD = 0.41, a moderate effect) and creatine (SMD = 0.30, a small to moderate effect) significantly enhance jump performance when combined with S&C training, with β-alanine exhibiting a slightly larger point estimate and a higher SUCRA value (89.0% vs. 76.1%). This suggests that for improving jump performance, both supplements are viable options, with β-alanine potentially offering a marginally greater average benefit in the context of the studies included. This finding is broadly consistent with a recent network meta-analysis in footballers, where both β-alanine and creatine also produced meaningful gains in vertical-jump height, though the reported effect sizes varied (6).

The ergogenic mechanisms of β-alanine and creatine are distinct yet complementary for high-intensity activities. β-alanine acts primarily by elevating intramuscular carnosine concentrations. Carnosine, a di-peptide of β-alanine and histidine, buffers intracellular H^+^, helping maintain acid–base balance and delaying fatigue during repeated high-intensity exercise bouts (49, 50). It may also heighten Ca^2+^ sensitivity and activate myosin ATPase, indirectly boosting explosive force (51). Creatine, by increasing readily available PCr, accelerates ATP resynthesis for immediate high-energy phosphate supply during anaerobic bursts (14). The observed moderate effect of β-alanine and small to moderate effect of creatine are plausible given these mechanisms and are supported by RCTs demonstrating improvements in power-oriented sports, especially when supplementation protocols (e.g., 4–6 weeks for β-alanine loading; creatine loading followed by maintenance) are appropriately followed (6, 47).

In contrast, protein (SMD = −0.16, a small negative effect, not statistically significant), vitamin D (SMD = −0.13, a negligible to small negative effect, not statistically significant), and caffeine (SMD = 0.18, a small effect, not statistically significant) did not significantly improve jump performance in our pooled analysis, and the certainty of these findings was ‘very low’. While vitamin D can modulate Ca^2+^ homeostasis and mitochondrial function via muscle VDRs (52), recent meta-analyses generally show no clear effect on maximal strength or jump height in athletes without pre-existing deficiency (53). Protein supplementation primarily supports chronic adaptations rather than acute jump enhancement. Although caffeine can acutely boost neuromuscular output through central stimulation (15, 54), and isolated trials have reported greater jump height with caffeine (55), the heterogeneity in dosing strategies (e.g., 3 vs 6 mg/kg/d in Wu et al. (69), individual tolerance and caffeine habituation, and varied study designs within our NMA likely contributed to the non-significant pooled outcome. Larger, well-controlled trials are needed to confirm any consistent benefit of these latter supplements on jump performance in athletes undergoing S&C.

### 4.4 Sprint speed

Our findings demonstrate that creatine supplementation, when combined with S&C, significantly enhances short-distance sprint performance. This was evidenced by a moderate effect (SMD = −0.42) favouring creatine over placebo, an effect supported by ‘moderate’ certainty evidence. This aligns well with the established mechanism whereby creatine elevates intramuscular PCr stores, thus reinforcing the phosphagen system during brief (<30s), maximal intensity efforts like sprinting (14). Previous research consistently reports that creatine improves both single- and repeated-sprint performance, likely due to faster ATP resynthesis, and potentially through enhanced force adaptation in fast-twitch fibres and heightened neuromuscular activation (56, 57).

By contrast, neither β-alanine (SMD = −0.23, a small effect favouring β-alanine, though not statistically significant) nor vitamin D (SMD = 0.13, a negligible to small effect favouring placebo, though not statistically significant) produced statistically significant gains in short sprints, and these findings were based on ‘very low’ certainty evidence. The small, non-significant trend observed for β-alanine might be because its primary performance benefit—enhancing intracellular buffering capacity against acidic metabolites (50) —is typically confined to high-intensity efforts of slightly longer duration (e.g., ≥60 s), where H^+^ accumulation becomes more limiting, rather than very brief sprints (58). Indeed, Jagim et al. (59) observed no improvement in intermittent-sprint tests after five weeks of β-alanine supplementation.

Although vitamin D modulates muscle physiology through skeletal-muscle VDRs, its direct effect on sprint speed remains unclear, especially in athletes with sufficient baseline vitamin D status. Recent systematic reviews note potential benefits for some aspects of anaerobic power, yet most RCTs report no change in sprint times (60). For example, Fairbairn et al. (61) found no difference in 30 m sprint time in rugby players with adequate baseline 25(OH)D levels after D₃ supplementation versus placebo. Thus, the lack of effect for β-alanine and vitamin D on sprint speed in our NMA likely reflects their primary mechanisms of action being less critical for the instantaneous energy turnover that dictates performance in very short-duration sprints (61, 62).

### 4.5 Muscle mass

Although HMB (SMD = 0.15), creatine (SMD = 0.16), and protein supplementation (SMD = 0.22) combined with S&C training exhibited small positive point estimates for effects on lean body mass (or muscle mass), none of these reached statistical significance in our NMA, as the 95% confidence intervals all crossed zero (*p* > 0.05). The certainty of these null or at best, only small and uncertain effects was ‘very low’ for all comparisons. These findings echo previous systematic reviews and meta-analyses. For instance, Sánchez-Martínez et al. (63) reported a trivial, non-significant effect of HMB on lean mass in trained athletes. Similarly, a meta-analysis by Burke et al. (64). More recently, Desai et al. (65), in a study involving a within-subject design with a supplement washout period rather than a parallel placebo group, observed no significant additional increase in fat-free mass (FFM) during a 12-week resistance training period with 5 g·day^−1^ creatine supplementation compared to training without supplementation. Interestingly, the authors reported changes in FFM even during the washout period, highlighting the complexities in interpreting FFM changes in such designs and the potential for carry-over effects or other influencing factors. Indicated that creatine supplementation combined with resistance training produces only a minute hypertrophic response. More recently, Desai et al. (65), in a study involving a supplement washout period rather than a parallel placebo group, observed no significant additional increase in fat-free mass (FFM) during a 12-week resistance training period with creatine supplementation compared to training without supplementation, noting changes in FFM even during the washout period. Protein supplements show a comparable pattern; while supplemental protein may contribute approximately 0.30 kg of additional lean mass over prolonged resistance training (66), these benefits tend to plateau once total daily protein intake exceeds optimal levels (e.g., ~1.6 g·kg^−1^·day^−1^) (37). In many of the studies included in our NMA, baseline protein intake was likely adequate (e.g., often reported around 1.2 g·kg^−1^·day^−1^ or higher) (42), which would attenuate any marginal gains from additional supplementation. Several factors may account for these non-significant findings regarding muscle mass. First, the duration of most interventions (typically <12 weeks) may have been insufficient to capture the slow accrual of significant myofibrillar hypertrophy, particularly in well-trained athletes who are often closer to their genetic adaptive ceiling. Second, heterogeneity in supplement form (e.g., calcium-salt versus free-acid HMB), dosage, and timing of intake, as well as variations in the S&C program intensity and volume, can cloud effect estimates. Third, as mentioned, a “ceiling effect” may apply when baseline protein intake is already optimal (37, 42). Finally, early weight gain associated with creatine is often attributed more to cell water retention rather than true contractile tissue hypertrophy; tangible gains in contractile protein may require longer or higher-dose protocols, or be more modest than popularly perceived (64, 65).

While HMB may show benefits in specific populations like sarcopenic or elderly individuals (67), its utility for muscle accretion in healthy, resistance-trained athletes remains questionable (66). Overall, current evidence from our NMA suggests that none of these three commonly used supplements confers a robust or statistically significant hypertrophic advantage in well-nourished, systematically trained athletes under typical training durations and dosing regimens investigated in the included studies.

### 4.6 Practical implications

Our systematic review and network meta-analysis indicate that different dietary supplements exert significant yet heterogeneous effects on muscular strength, explosive power, and speed in athletes. This finding underscores the need for coaches and sport-nutrition practitioners to tailor supplementation protocols to both sport-specific demands and individual athlete characteristics. During training phases aimed at maximising strength, priority should be given to adequate, high-quality protein intake to support muscle protein synthesis and repair. For brief, high-intensity actions such as jumping, creatine and β-alanine are both effective; β-alanine’s superior buffering capacity can, in certain contexts, prolong peak force generation and thus improve jump height and consistency. In sprinting and other phosphocreatine-dependent efforts, creatine supplementation is likely more advantageous for rapid energy release and acceleration. In hybrid sports requiring concurrent strength and power (e.g., basketball, rugby, weightlifting), baseline protein intake should be secured before combining β-alanine with creatine to achieve synergistic, multidimensional performance gains. Critically, all nutritional interventions must be integrated within a periodised training framework and iteratively adjusted on the basis of periodic monitoring, thereby maximising the synergy among training, nutrition, and recovery while ensuring athlete safety.

### 4.7 Strengths and limitations

The principal strength of the present work is its status as the most comprehensive synthesis to date of how dietary supplements, in conjunction with physical-conditioning programmes, affect multiple performance dimensions in systematically trained athletes—maximal strength, jump performance, sprint speed, and lean body mass. By incorporating both direct head-to-head trial evidence and indirect comparisons through a NMA, this study offers a broader comparative assessment and ranking of competing interventions, which provides high decision-making value for practitioners. Methodologically, we applied the minimally contextualized framework within GRADE, For judging imprecision, in the absence of established minimally clinical important differences (MCIDs) for all our outcome measures in athletes, we primarily considered whether the confidence interval for an effect estimate crossed the line of no effect (null value) as a critical threshold for imprecision, a common practice in evidence synthesis when specific MCIDs are undefined (as detailed in our Methods section and Appendix S1). A further innovation is the integration of S&C outcomes with nutritional interventions, yielding multidimensional evidence that can inform personalised, integrated training–nutrition strategies for coaches and sports dietitians.

Notwithstanding these strengths, several limitations warrant attention. The included trials varied considerably in supplement type, dosage, intervention duration, and strength and conditioning protocols, complicating the interpretation and synthesis of pooled effects. Moreover, just 3 of the 35 RCTs were judged at low risk of bias, so the pooled effects may be inflated or attenuated. Most pairwise comparisons were based on low- or very low-certainty evidence, largely due to methodological concerns in the underlying RCTs, imprecise effect estimates, and—in some cases—evidence of heterogeneity or network inconsistency. As such, the conclusions drawn should be interpreted with caution. Sex imbalance was another concern, with male participants substantially outnumbering females, limiting the generalisability of findings to female athletes. Moreover, only a limited number of small-scale RCTs evaluated supplements such as HMB, vitamin D, and other less-studied compounds, weakening the stability of indirect comparisons and contributing to uncertainty in the network estimates. Future studies should adopt standardised supplementation and training protocols, harmonise measurement techniques, balance sex representation, and employ longer follow-ups to enhance the reliability and applicability of the evidence.

## 5 Conclusion

This network meta-analysis indicates that protein supplementation is the most effective strategy for enhancing muscular strength, whereas both β-alanine and creatine significantly improve jump performance—with β-alanine displaying a marginally superior effect size and SUCRA ranking. Creatine shows the clearest advantage for accelerating 30-m sprint speed, yet none of the supplements produced a statistically significant gain in muscle mass. Practically, athletes should prioritise high-quality protein during strength-focused phases; employ a combined β-alanine–creatine protocol when training for explosive power; and emphasise creatine during sprint-specific work to optimise the synergy between training, nutrition and performance. Coaches and sports nutritionists are encouraged to design periodised, sport-specific and individualised supplementation plans to maximise athletic outcomes.

## Data Availability

The original contributions presented in the study are included in the article/Supplementary material, further inquiries can be directed to the corresponding authors.
