# Correction: Effects of different dietary supplements combined with conditioning training on muscle strength, jump performance, sprint speed, and muscle mass in athletes: a systematic review and network meta-analysis

**DOI:** 10.3389/fnut.2025.1670394

**Published:** 2025-08-05

**Authors:** Beiwang Deng, Ruixiang Yan, Tianyuan He, Gesheng Lin, Ting Liu, Wen Chen, Jiaxin He, Duanying Li

**Affiliations:** ^1^School of Athletic Training, Guangzhou Sport University, Guangzhou, Guangdong, China; ^2^Sports Industry Department, Guangzhou Polytechnic of Sports, Guangzhou, China; ^3^Department of Physical Education, Guangzhou Sport University, Guangzhou, China; ^4^Guangdong Provincial Key Laboratory of Human Sports Performance Science, Guangzhou Sport University, Guangzhou, Guangdong, China

**Keywords:** sport nutrition, ergogenic aids, supplementation, sport performance, sportsman

In the published article, there was an error in the **Affiliations**. The affiliation for authors Dr. Jiaxin He and Dr. Duanying Li was incorrectly given as “Department of Physical Education, Guangzhou Sport University, Guangzhou, China”. The correct affiliation is “School of Athletic Training, Guangzhou Sport University, Guangzhou, Guangdong, China”.

In addition, the following affiliation has been added for author Dr. Duanying Li: “Guangdong Provincial Key Laboratory of Human Sports Performance Science, Guangzhou Sport University, Guangzhou, Guangdong, China”.

The original version of this article has been updated.

