# Supplementary material for: Effects of different dietary supplements combined with conditioning training on muscle strength, jump performance, sprint speed, and muscle mass in athletes: a systematic review and network meta-analysis
Source: Front Nutr. 2025 Jul 9;12:1636970. doi: 10.3389/fnut.2025.1636970 (PMC12295849; doi:10.3389/fnut.2025.1636970)
Supplement: Supplementary file 1 [file Table_1.docx]

Supplementary Appendix

**Table of contents**

[List of Abbreviations 2](#_Toc20238)

[Appendix 1: Search strategy 3](#_Toc17788)

[Appendix 2: Characteristics of included studies 12](#_Toc1311)

[Appendix 3: Risk of bias of randomized clinical trials 15](#_Toc3484)

[Appendix 4: Evaluation of inconsistency and heterogeneity 17](#_Toc19816)

[Appendix 5: Network maps and forest plots of secondary outcomes 18](#_Toc13297)

[Appendix 6: SUCRA and cumulative probability plots 22](#_Toc14716)

[Appendix 7: League Table of Summary Estimates for Dietary Supplementation Combined with Strength and Conditioning on Athletic Performance from on Network Meta-Analysis 23](#_Toc15513)

[Appendix 8: CINeMA Assessment 25](#_Toc31351)

[Figure S8.3: Risk of bias contribution by intervention group in jumping performance 27](#_Toc9195)

[Appendix 9: Funnel plots 33](#_Toc5208)

[Appendix S1 : Detailed CINeMA Assessment Protocol 35](#_Toc31459)

# List of Abbreviations

The following abbreviations are used in this manuscript:

| **Abbreviation** | **Full Term** |
| --- | --- |
| HMB | β-hydroxy-β-methylbutyrate |
| S&C | strength and conditioning |
| CINeMA | Confidence in Network Meta-Analysis |
| SUCRA | surface under the cumulative ranking curve |
| PL | placebo |
| PR | protein |
| CR | creatine |
| BA | β-alanine |
| VD | Vitamin D3 |
| CAF | caffeine |

# Appendix 1: Search strategy

**Table S1.** Search strategy of Pubmed

| **#** | **Searches** |
| --- | --- |
| 1 | ((((((((((((((((((((((((((((((((((((((((((((((((((Athlete[MeSH Terms]) OR (athlete[Title/Abstract])) OR (Professional Athletes[Title/Abstract])) OR (Athlete, Professional[Title/Abstract])) OR (Athletes, Professional[Title/Abstract])) OR (Professional Athlete[Title/Abstract])) OR (Elite Athletes[Title/Abstract])) OR (Athlete, Elite[Title/Abstract])) OR (Athletes, Elite[Title/Abstract])) OR (Elite Athlete[Title/Abstract])) OR (College Athletes[Title/Abstract])) OR (Athlete, College[Title/Abstract])) OR (Athletes, College[Title/Abstract])) OR (College Athlete[Title/Abstract])) OR (sports playe[Title/Abstract])) OR (Sportsperson[Title/Abstract])) OR (trained individuals[Title/Abstract])) OR (Sports[Title/Abstract])) OR (Football[Title/Abstract])) OR (Soccer[Title/Abstract])) OR (hockey[Title/Abstract])) OR (basketball[Title/Abstract])) OR (Netball[Title/Abstract])) OR (Volleyball[Title/Abstract])) OR (track and field[Title/Abstract])) OR (Cycli[Title/Abstract])) OR (running[Title/Abstract])) OR (Runner[Title/Abstract])) OR (Swim[Title/Abstract])) OR (Handball[Title/Abstract])) OR (Softball[Title/Abstract])) OR (Tennis[Title/Abstract])) OR (baseball[Title/Abstract])) OR (cross country[Title/Abstract])) OR (cricket[Title/Abstract])) OR (Surf[Title/Abstract])) OR (Skiing[Title/Abstract])) OR (Golf[Title/Abstract])) OR (Hurdling[Title/Abstract])) OR (Bicycling[Title/Abstract])) OR (Boxing[Title/Abstract])) OR (Gymnast[Title/Abstract])) OR (martial arts[Title/Abstract])) OR (racquet sports[Title/Abstract])) OR (Badminton[Title/Abstract])) OR (Jogg[Title/Abstract])) OR (Walk[Title/Abstract])) OR (weight lifting[Title/Abstract])) OR (Lift[Title/Abstract])) OR (Weights[Title/Abstract])) OR (wrestling[Title/Abstract]) |
| 2 | ((((((((((((((((((((((((((((((((((((((((((((((((((((Dietary Supplement[MeSH Terms]) OR (nutrition intervention,[Title/Abstract])) OR (nutrition supplement[Title/Abstract])) OR (nutrition support[Title/Abstract])) OR (nutrition therapy[Title/Abstract])) OR (nutrition method[Title/Abstract])) OR (nutrition technique[Title/Abstract])) OR (nutrition treatment[Title/Abstract])) OR (nutrition modification[Title/Abstract])) OR (multinutrient supplement[Title/Abstract])) OR (multinutrient intervention[Title/Abstract])) OR (nutritional supplementation[Title/Abstract])) OR (Supplements, Dietary[Title/Abstract])) OR (Dietary Supplementations[Title/Abstract])) OR (Supplementations, Dietary[Title/Abstract])) OR (Nutrition Therapy[Title/Abstract])) OR (Food Supplementations[Title/Abstract])) OR (Food Supplements[Title/Abstract])) OR (Food Supplement[Title/Abstract])) OR (Supplement, Food[Title/Abstract])) OR (Supplements, Food[Title/Abstract])) OR (Nutraceuticals[Title/Abstract])) OR (Nutraceutical[Title/Abstract])) OR (Nutriceuticals[Title/Abstract])) OR (Nutriceutical[Title/Abstract])) OR (Neutraceuticals[Title/Abstract])) OR (Neutraceutical[Title/Abstract])) OR (Herbal Supplements[Title/Abstract])) OR (Herbal Supplement[Title/Abstract])) OR (Supplement, Herbal[Title/Abstract])) OR (Supplements, Herbal[Title/Abstract])) OR (nutritional intake[Title/Abstract])) OR (protein supplement[Title/Abstract])) OR (Creatine[Title/Abstract])) OR (beta Alanine[Title/Abstract])) OR (β-alanine[Title/Abstract])) OR (Leucine[Title/Abstract])) OR (amino acid[Title/Abstract])) OR (carbohydrate loading[Title/Abstract])) OR (branched chain amino acid[Title/Abstract])) OR (proteins[Title/Abstract])) OR (protein[Title/Abstract])) OR (amino acids[Title/Abstract])) OR (amino[Title/Abstract])) OR (acids[Title/Abstract])) OR (leucine[Title/Abstract])) OR (whey proteins[Title/Abstract])) OR (whey protein[Title/Abstract])) OR (whey[Title/Abstract])) OR (vitamin[Title/Abstract])) OR (beetroot juice[Title/Abstract])) OR (caffeine[Title/Abstract])) OR (HMB[Title/Abstract]) |
| 3 | ((((((((((((((((((((((((((((((((Physical Conditioning, Human[MeSH Terms]) OR (Fitness Training[Title/Abstract])) OR (Athletic Conditioning[Title/Abstract])) OR (Strength AND Conditioning[Title/Abstract])) OR (Sport Training[Title/Abstract])) OR (Motor Control Training[Title/Abstract])) OR (Functional Training[Title/Abstract])) OR (Functional Training[Title/Abstract])) OR (Resistance Training[Title/Abstract])) OR (Resistance Training[Title/Abstract])) OR (Resistance Training[Title/Abstract])) OR (Resistance Training[Title/Abstract])) OR (Training, Resistance[Title/Abstract])) OR (Weight Training[Title/Abstract])) OR (Weight-Lifting Strengthening Program[Title/Abstract])) OR (Strengthening Program, Weight-Lifting[Title/Abstract])) OR (Weight Lifting Strengthening Program[Title/Abstract])) OR (Weight-Lifting Strengthening Programs[Title/Abstract])) OR (Weight-Lifting Exercise Program[Title/Abstract])) OR (Exercise Programs, Weight-Lifting[Title/Abstract])) OR (Exercise Program, Weight-Lifting[Title/Abstract])) OR (Weight Lifting Exercise Program[Title/Abstract])) OR (Weight-Lifting Exercise Programs[Title/Abstract])) OR (Weight-Bearing Strengthening Program[Title/Abstract])) OR (Strengthening Programs, Weight-Bearing[Title/Abstract])) OR (Strengthening Program, Weight-Bearing[Title/Abstract])) OR (Weight Bearing Strengthening Program[Title/Abstract])) OR (Weight-Bearing Strengthening Programs[Title/Abstract])) OR (Weight-Bearing Exercise Program[Title/Abstract])) OR (Exercise Programs, Weight-Bearing[Title/Abstract])) OR (Exercise Program, Weight-Bearing[Title/Abstract])) OR (Weight Bearing Exercise Program[Title/Abstract])) OR (Weight-Bearing Exercise Programs[Title/Abstract]) |
| 4 | ((((((((((((((((((((((((((((((((((((((Plyometric Training[Title/Abstract]) OR (Plyometric Exercises[Title/Abstract])) OR (Plyometric Drills[Title/Abstract])) OR (Exercise, Plyometric[Title/Abstract])) OR (Exercises, Plyometric[Title/Abstract])) OR (Training, Plyometric[Title/Abstract])) OR (Trainings, Plyometric[Title/Abstract])) OR (Stretch-Shortening Cycle[Title/Abstract])) OR (Stretch-Shortening Exercises[Title/Abstract])) OR (Stretch-Shortening Drills[Title/Abstract])) OR (Stretch-Shortening Exercise[Title/Abstract])) OR (Stretch-Shortening Cycle Exercise[Title/Abstract])) OR (Stretch-Shortening Cycle Exercise[Title/Abstract])) OR (Stretch Shortening Exercise[Title/Abstract])) OR (Stretch Shortening Drill[Title/Abstract])) OR (Drill, Plyometric[Title/Abstract])) OR (Drills, Plyometric[Title/Abstract])) OR (Drill, Stretch-Shortening[Title/Abstract])) OR (Drills, Stretch-Shortening[Title/Abstract])) OR (Endurance Training[Title/Abstract])) OR (Aerobic Training[Title/Abstract])) OR (Sprint Interval Training[Title/Abstract])) OR (Sprint Training[Title/Abstract])) OR (Sprint Training[Title/Abstract])) OR (Agility Training[Title/Abstract])) OR (High-Intensity Interval Training[Title/Abstract])) OR (HIIT[Title/Abstract])) OR (Interval Training, High-Intensity[Title/Abstract])) OR (Interval Trainings, High-Intensity[Title/Abstract])) OR (Training, High-Intensity Interval[Title/Abstract])) OR (Training, High-Intensity Interval[Title/Abstract])) OR (Training, High-Intensity Interval[Title/Abstract])) OR (Exercise, High-Intensity Intermittent[Title/Abstract])) OR (Exercises, High-Intensity Intermittent[Title/Abstract])) OR (High-Intensity Intermittent Exercises[Title/Abstract])) OR (Anaerobic Training[Title/Abstract])) OR (Anaerobic Exercise[Title/Abstract])) OR (Anaerobic Capacity[Title/Abstract])) OR (Sprint Exercise[Title/Abstract]) |
| 5 | ((((((randomized controlled trial[Publication Type]) OR (randomized)) OR (clinical trials)) OR (placebo)) OR (randomly)) OR (trial)) OR (RCT) |
| 6 | #3 OR #4 |
| 7 | #1 AND #2 AND #5 AND #6 |

**Table S2.** Search strategy of Web of Science

| **#** | **Searches** |
| --- | --- |
| 1 | TS=("Athlete" OR "athlete" OR "Professional Athletes" OR "Athlete, Professional" OR "Athletes, Professional" OR "Professional Athlete" OR "Elite Athletes" OR "Athlete, Elite" OR "Athletes, Elite" OR "Athletes, Elite" OR "College Athletes" OR "College Athletes" OR "Athlete, College" OR "Athletes, College" OR "College Athlete" OR "sports playe" OR "Sportsperson" OR "trained individuals" OR "Sports" OR "Football" OR "Soccer" OR "hockey" OR "basketball" OR "Netball" OR "Volleyball" OR "track and field" OR "Cycli" OR "running" OR "Runner" OR "Swim" OR "Handball" OR "Softball" OR "Tennis" OR "baseball" OR "cross country" OR "cricket" OR "Surf" OR "Skiing" OR "Golf" OR "Hurdling" OR "Bicycling" OR "Boxing" OR "Gymnast" OR "martial arts" OR "racquet sports" OR "Badminton" OR "Jogg" OR "Walk" OR "weight lifting" OR "Lift" OR "Weights" OR "wrestling") |
| 2 | TS=("Dietary Supplement" OR "nutrition intervention" OR "nutrition supplement" OR "nutrition support" OR "nutrition therapy" OR "nutrition method" OR "nutrition technique" OR "nutrition treatment" OR "nutrition modification" OR "multinutrient supplement" OR "multinutrient intervention" OR "nutritional supplementation" OR "Supplements, Dietary" OR "Dietary Supplementations" OR "Supplementations, Dietary" OR "Nutrition Therapy" OR "Food Supplementations" OR "Food Supplements" OR "Food Supplement" OR "Supplement, Food" OR "Supplements, Food" OR "Nutraceuticals" OR "Nutraceutical" OR "Nutriceuticals" OR "Nutriceutical" OR "Neutraceuticals" OR "Neutraceutical" OR "Herbal Supplements" OR "Herbal Supplement" OR "Supplement, Herbal" OR "Supplements, Herbal" OR "nutritional intake" OR "protein supplement" OR "Creatine" OR "beta Alanine" OR "β-alanine" OR "Leucine" OR "amino acid" OR "carbohydrate loading" OR "branched chain amino acid" OR "proteins" OR "protein" OR "amino acids" OR "amino" OR "acids" OR "leucine" OR "whey proteins" OR "whey protein" OR "whey" OR "vitamin" OR "beetroot juice" OR "caffeine" OR "HMB") |
| 3 | TS=("Physical Conditioning, Human" OR "Fitness Training" OR "Athletic Conditioning" OR "Strength AND Conditioning" OR "Sport Training" OR "Motor Control Training" OR "Functional Training" OR "Resistance Training" OR "Training, Resistance" OR "Weight Training" OR "Weight-Lifting Strengthening Program" OR "Strengthening Program, Weight-Lifting" OR "Weight Lifting Strengthening Program" OR "Weight-Lifting Strengthening Programs" OR "Weight-Lifting Exercise Program" OR "Exercise Programs, Weight-Lifting" OR "Exercise Program, Weight-Lifting" OR "Weight Lifting Exercise Program" OR "Weight-Lifting Exercise Programs" OR "Weight-Bearing Strengthening Program" OR "Strengthening Programs, Weight-Bearing" OR "Strengthening Program, Weight-Bearing" OR "Weight Bearing Strengthening Program" OR "Weight-Bearing Strengthening Programs" OR "Weight-Bearing Exercise Program" OR "Exercise Programs, Weight-Bearing" OR "Exercise Program, Weight-Bearing" OR "Weight Bearing Exercise Program" OR "Weight-Bearing Exercise Programs") |
| 4 | TS=("Plyometric Training" OR "Plyometric Exercises" OR "Plyometric Drills" OR "Exercise, Plyometric" OR "Exercises, Plyometric" OR "Training, Plyometric" OR "Trainings, Plyometric" OR "Stretch-Shortening Cycle" OR "Stretch-Shortening Exercises" OR "Stretch-Shortening Drills" OR "Stretch-Shortening Exercise" OR "Stretch-Shortening Cycle Exercise" OR "Stretch Shortening Exercise" OR "Stretch Shortening Drill" OR "Drill, Plyometric" OR "Drills, Plyometric" OR "Drill, Stretch-Shortening" OR "Drills, Stretch-Shortening" OR "Endurance Training" OR "Aerobic Training" OR "Sprint Interval Training" OR "Sprint Training" OR "Agility Training" OR "High-Intensity Interval Training" OR "HIIT" OR "Interval Training, High-Intensity" OR "Interval Trainings, High-Intensity" OR "Training, High-Intensity Interval" OR "Exercise, High-Intensity Intermittent" OR "Exercises, High-Intensity Intermittent" OR "High-Intensity Intermittent Exercises" OR "Anaerobic Training" OR "Anaerobic Exercise" OR "Anaerobic Capacity" OR "Sprint Exercise") |
| 5 | ALL=("randomized controlled trial" OR "randomized" OR "clinical trials" OR "placebo" OR "randomly" OR "trial" OR "RCT") |
| 6 | #3 AND #4 |
| 7 | #1 AND #2 AND #5 AND #6 |

**Table S3.** Search strategy of Embase

| **#** | **Searches** |
| --- | --- |
| 1 | 'athlete'/exp |
| 2 | athlete:ti,ab,kw OR 'professional athlete':ti,ab,kw OR 'elite athlete':ti,ab,kw OR 'collegiate athlete':ti,ab,kw OR 'sports player':ti,ab,kw OR 'sportsperson':ti,ab,kw OR 'trained individuals':ti,ab,kw OR 'resistance-trained':ti,ab,kw OR 'strength-trained':ti,ab,kw OR 'power-trained':ti,ab,kw OR 'weightlifter':ti,ab,kw OR 'powerlifter':ti,ab,kw OR 'strength athlete':ti,ab,kw OR 'bodybuilder':ti,ab,kw OR 'endurance athlete':ti,ab,kw OR 'team-sport athlete':ti,ab,kw OR 'high-performance athlete':ti,ab,kw OR 'physically trained individuals':ti,ab,kw OR 'competitive athlete':ti,ab,kw OR 'football':ti,ab,kw OR 'soccer':ti,ab,kw OR 'hockey':ti,ab,kw OR 'basketball':ti,ab,kw OR 'netball':ti,ab,kw OR 'volleyball':ti,ab,kw OR 'track and field':ti,ab,kw OR 'cycling':ti,ab,kw OR 'running':ti,ab,kw OR 'runner':ti,ab,kw OR 'swimming':ti,ab,kw OR 'swimmer':ti,ab,kw OR 'handball':ti,ab,kw OR 'softball':ti,ab,kw OR 'tennis':ti,ab,kw OR 'baseball':ti,ab,kw OR 'cross country':ti,ab,kw OR 'cricket':ti,ab,kw OR 'surfing':ti,ab,kw OR 'skiing':ti,ab,kw OR 'golf':ti,ab,kw OR 'hurdling':ti,ab,kw OR 'bicycling':ti,ab,kw OR 'boxing':ti,ab,kw OR 'gymnastics':ti,ab,kw OR 'martial arts':ti,ab,kw OR 'racquet sports':ti,ab,kw OR 'badminton':ti,ab,kw OR 'jogging':ti,ab,kw OR 'walking':ti,ab,kw OR 'weight lifting':ti,ab,kw OR 'weight training':ti,ab,kw OR 'wrestling':ti,ab,kw |
| 3 | 'dietary supplement'/exp |
| 4 | 'nutrition intervention':ti,ab,kw OR 'nutrition supplement':ti,ab,kw OR 'nutrition support':ti,ab,kw OR 'nutrition method':ti,ab,kw OR 'nutrition technique':ti,ab,kw OR 'nutrition treatment':ti,ab,kw OR 'nutrition modification':ti,ab,kw OR 'multinutrient supplement':ti,ab,kw OR 'multinutrient intervention':ti,ab,kw OR 'nutritional supplementation':ti,ab,kw OR 'supplements, dietary':ti,ab,kw OR 'dietary supplementations':ti,ab,kw OR 'supplementations, dietary':ti,ab,kw OR 'nutrition therapy':ti,ab,kw OR 'food supplementations':ti,ab,kw OR 'food supplements':ti,ab,kw OR 'food supplement':ti,ab,kw OR 'supplement, food':ti,ab,kw OR 'supplements, food':ti,ab,kw OR 'nutraceuticals':ti,ab,kw OR 'nutraceutical':ti,ab,kw OR 'nutriceuticals':ti,ab,kw OR 'nutriceutical':ti,ab,kw OR 'neutraceuticals':ti,ab,kw OR 'neutraceutical':ti,ab,kw OR 'herbal supplements':ti,ab,kw OR 'herbal supplement':ti,ab,kw OR 'supplement, herbal':ti,ab,kw OR 'supplements, herbal':ti,ab,kw OR 'nutritional intake':ti,ab,kw OR 'protein supplement':ti,ab,kw OR 'creatine':ti,ab,kw OR 'beta alanine':ti,ab,kw OR 'β-alanine':ti,ab,kw OR 'leucine':ti,ab,kw OR 'amino acid':ti,ab,kw OR 'carbohydrate loading':ti,ab,kw OR 'branched chain amino acid':ti,ab,kw OR 'proteins':ti,ab,kw OR 'protein':ti,ab,kw OR 'amino acids':ti,ab,kw OR 'amino':ti,ab,kw OR 'acids':ti,ab,kw OR 'whey proteins':ti,ab,kw OR 'whey protein':ti,ab,kw OR 'whey':ti,ab,kw OR 'vitamin':ti,ab,kw OR 'beetroot juice':ti,ab,kw OR 'caffeine':ti,ab,kw OR 'hmb':ti,ab,kw |
| 5 | 'physical conditioning, human'/exp |
| 6 | 'physical conditioning, human':ti,ab,kw OR 'fitness training':ti,ab,kw OR 'athletic conditioning':ti,ab,kw OR ('strength':ti,ab,kw AND 'conditioning':ti,ab,kw) OR 'sport training':ti,ab,kw OR 'motor control training':ti,ab,kw OR 'functional training':ti,ab,kw OR 'resistance training':ti,ab,kw OR 'training, resistance':ti,ab,kw OR 'weight training':ti,ab,kw OR 'weight-lifting strengthening program':ti,ab,kw OR 'strengthening program, weight-lifting':ti,ab,kw OR 'weight lifting strengthening program':ti,ab,kw OR 'weight-lifting strengthening programs':ti,ab,kw OR 'weight-lifting exercise program':ti,ab,kw OR 'exercise programs, weight-lifting':ti,ab,kw OR 'exercise program, weight-lifting':ti,ab,kw OR 'weight lifting exercise program':ti,ab,kw OR 'weight-lifting exercise programs':ti,ab,kw OR 'weight-bearing strengthening program':ti,ab,kw OR 'strengthening programs, weight-bearing':ti,ab,kw OR 'strengthening program, weight-bearing':ti,ab,kw OR 'weight bearing strengthening program':ti,ab,kw OR 'weight-bearing strengthening programs':ti,ab,kw OR 'weight-bearing exercise program':ti,ab,kw OR 'exercise programs, weight-bearing':ti,ab,kw OR 'exercise program, weight-bearing':ti,ab,kw OR 'weight bearing exercise program':ti,ab,kw OR 'weight-bearing exercise programs':ti,ab,kw |
| 7 | 'plyometric training':ti,ab,kw OR 'plyometric exercises':ti,ab,kw OR 'plyometric drills':ti,ab,kw OR 'exercise, plyometric':ti,ab,kw OR 'exercises, plyometric':ti,ab,kw OR 'training, plyometric':ti,ab,kw OR 'trainings, plyometric':ti,ab,kw OR 'stretch-shortening cycle':ti,ab,kw OR 'stretch-shortening exercises':ti,ab,kw OR 'stretch-shortening drills':ti,ab,kw OR 'stretch-shortening exercise':ti,ab,kw OR 'stretch-shortening cycle exercise':ti,ab,kw OR 'stretch shortening exercise':ti,ab,kw OR 'stretch shortening drill':ti,ab,kw OR 'drill, plyometric':ti,ab,kw OR 'drills, plyometric':ti,ab,kw OR 'drill, stretch-shortening':ti,ab,kw OR 'drills, stretch-shortening':ti,ab,kw OR 'endurance training':ti,ab,kw OR 'aerobic training':ti,ab,kw OR 'sprint interval training':ti,ab,kw OR 'sprint training':ti,ab,kw OR 'agility training':ti,ab,kw OR 'high-intensity interval training':ti,ab,kw OR 'hiit':ti,ab,kw OR 'interval training, high-intensity':ti,ab,kw OR 'interval trainings, high-intensity':ti,ab,kw OR 'training, high-intensity interval':ti,ab,kw OR 'exercise, high-intensity intermittent':ti,ab,kw OR 'exercises, high-intensity intermittent':ti,ab,kw OR 'high-intensity intermittent exercises':ti,ab,kw OR 'anaerobic training':ti,ab,kw OR 'anaerobic exercise':ti,ab,kw OR 'anaerobic capacity':ti,ab,kw OR 'sprint exercise':ti,ab,kw |
| 8 | 'randomized controlled trial'/exp |
| 9 | 'randomized controlled trial':ti,ab,kw OR 'randomized':ti,ab,kw OR 'clinical trials':ti,ab,kw OR 'placebo':ti,ab,kw OR 'randomly':ti,ab,kw OR 'trial':ti,ab,kw OR 'rct':ti,ab,kw |
| 10 | #1 OR #2 |
| 11 | #3 OR #4 |
| 12 | #5 OR #6 OR #7 |
| 13 | #8 OR #9 |
| 14 | #10 AND #11 AND #12 AND #13 |

**Table S4.** Search strategy of SPORTDiscus

| **#** | **Searches** |
| --- | --- |
| 1 | SU "Athlete" OR "athlete" OR "Professional Athletes" OR "Athlete, Professional" OR "Athletes, Professional" OR "Professional Athlete" OR "Elite Athletes" OR "Athlete, Elite" OR "Athletes, Elite" OR "Athletes, Elite" OR "College Athletes" OR "College Athletes" OR "Athlete, College" OR "Athletes, College" OR "College Athlete" OR "sports playe" OR "Sportsperson" OR "trained individuals" OR "Sports" OR "Football" OR "Soccer" OR "hockey" OR "basketball" OR "Netball" OR "Volleyball" OR "track and field" OR "Cycli" OR "running" OR "Runner" OR "Swim" OR "Handball" OR "Softball" OR "Tennis" OR "baseball" OR "cross country" OR "cricket" OR "Surf" OR "Skiing" OR "Golf" OR "Hurdling" OR "Bicycling" OR "Boxing" OR "Gymnast" OR "martial arts" OR "racquet sports" OR "Badminton" OR "Jogg" OR "Walk" OR "weight lifting" OR "Lift" OR "Weights" OR "wrestling" |
| 2 | SU "Dietary Supplement" OR "nutrition intervention" OR "nutrition supplement" OR "nutrition support" OR "nutrition therapy" OR "nutrition method" OR "nutrition technique" OR "nutrition treatment" OR "nutrition modification" OR "multinutrient supplement" OR "multinutrient intervention" OR "nutritional supplementation" OR "Supplements, Dietary" OR "Dietary Supplementations" OR "Supplementations, Dietary" OR "Nutrition Therapy" OR "Food Supplementations" OR "Food Supplements" OR "Food Supplement" OR "Supplement, Food" OR "Supplements, Food" OR "Nutraceuticals" OR "Nutraceutical" OR "Nutriceuticals" OR "Nutriceutical" OR "Neutraceuticals" OR "Neutraceutical" OR "Herbal Supplements" OR "Herbal Supplement" OR "Supplement, Herbal" OR "Supplements, Herbal" OR "nutritional intake" OR "protein supplement" OR "Creatine" OR "beta Alanine" OR "β-alanine" OR "Leucine" OR "amino acid" OR "carbohydrate loading" OR "branched chain amino acid" OR "proteins" OR "protein" OR "amino acids" OR "amino" OR "acids" OR "leucine" OR "whey proteins" OR "whey protein" OR "whey" OR "vitamin" OR "beetroot juice" OR "caffeine" OR "HMB" |
| 3 | SU "Physical Conditioning, Human" OR "Fitness Training" OR "Athletic Conditioning" OR "Strength and Conditioning" OR "Sport Training" OR "Motor Control Training" OR "Functional Training" OR "Resistance Training" OR "Training, Resistance" OR "Weight Training" OR "Weight-Lifting Strengthening Program" OR "Strengthening Programs, Weight-Lifting" OR "Strengthening Program, Weight-Lifting" OR "Weight Lifting Strengthening Program" OR "Weight-Lifting Strengthening Programs" OR "Weight-Lifting Exercise Program" OR "Exercise Programs, Weight-Lifting" OR "Exercise Program, Weight-Lifting" OR "Weight Lifting Exercise Program" OR "Weight-Lifting Exercise Programs" OR "Weight-Bearing Strengthening Program" OR "Strengthening Programs, Weight-Bearing" OR "Strengthening Program, Weight-Bearing" OR "Weight Bearing Strengthening Program" OR "Weight-Bearing Strengthening Programs" OR "Weight-Bearing Exercise Program" OR "Exercise Programs, Weight-Bearing" OR "Exercise Program, Weight-Bearing" OR "Weight Bearing Exercise Program" OR "Weight-Bearing Exercise Programs" |
| 4 | "Plyometric Training" OR "Plyometric Exercises" OR "Plyometric Drills" OR "Exercise, Plyometric" OR "Exercises, Plyometric" OR "Training, Plyometric" OR "Trainings, Plyometric" OR "Stretch-Shortening Cycle" OR "Stretch-Shortening Exercises" OR "Stretch-Shortening Drills" OR "Stretch-Shortening Exercise" OR "Stretch-Shortening Cycle Exercise" OR "Stretch Shortening Exercise" OR "Stretch Shortening Drill" OR "Drill, Plyometric" OR "Drills, Plyometric" OR "Drill, Stretch-Shortening" OR "Drills, Stretch-Shortening" OR "Endurance Training" OR "Aerobic Training" OR "Sprint Interval Training" OR "Sprint Training" OR "Agility Training" OR "High-Intensity Interval Training" OR "HIIT" OR "Interval Training, High-Intensity" OR "Interval Trainings, High-Intensity" OR "Exercise, High-Intensity Intermittent" OR "Exercises, High-Intensity Intermittent" OR "High-Intensity Intermittent Exercises" OR "Anaerobic Training" OR "Anaerobic Exercise" OR "Anaerobic Capacity" OR "Sprint Exercise" |
| 5 | TX "randomized controlled trial" OR "randomized" OR "clinical trials" OR "placebo" OR "randomly" OR "trial" OR "RCT" |
| 6 | #3 OR #4 |
| 7 | #1 AND #2 AND #5 AND #6 |

**Table S5.** Search strategy of Cochrane library

| **#** | **Searches** |
| --- | --- |
| 1 | MeSH descriptor: [Athletes] explode all trees |
| 2 | ('athlete' OR 'professional athlete' OR 'elite athlete' OR 'collegiate athlete' OR 'sports player' OR 'sportsperson' OR 'trained individuals' OR 'resistance-trained' OR 'strength-trained' OR 'power-trained' OR 'weightlifter' OR 'powerlifter' OR 'strength athlete' OR 'bodybuilder' OR 'endurance athlete' OR 'team-sport athlete' OR 'high-performance athlete' OR 'physically trained individuals' OR 'competitive athlete' OR 'football' OR 'soccer' OR 'hockey' OR 'basketball' OR 'netball' OR 'volleyball' OR 'track and field' OR 'cycling' OR 'running' OR 'runner' OR 'swimming' OR 'swimmer' OR 'handball' OR 'softball' OR 'tennis' OR 'baseball' OR 'cross country' OR 'cricket' OR 'surfing' OR 'skiing' OR 'golf' OR 'hurdling' OR 'bicycling' OR 'boxing' OR 'gymnastics' OR 'martial arts' OR 'racquet sports' OR 'badminton' OR 'jogging' OR 'walking' OR 'weight lifting' OR 'weight training' OR 'wrestling'):ti,ab,kw |
| 3 | MeSH descriptor: [Dietary Supplements] explode all trees |
| 4 | ('dietary supplement' OR 'nutrition supplement' OR 'nutrition support' OR 'nutrition method' OR 'nutrition technique' OR 'nutrition treatment' OR 'nutrition modification' OR 'multinutrient supplement' OR 'multinutrient intervention' OR 'nutrition intervention' OR 'supplements, dietary' OR 'dietary supplementations' OR 'supplementations, dietary' OR 'nutrition therapy' OR 'food supplementations' OR 'food supplements' OR 'food supplement' OR 'supplement, food' OR 'supplements, food' OR 'nutraceuticals' OR 'nutraceutical' OR 'nutriceuticals' OR 'nutriceutical' OR 'neutraceuticals' OR 'neutraceutical' OR 'herbal supplements' OR 'herbal supplement' OR 'supplement, herbal' OR 'supplements, herbal' OR 'nutritional intake' OR 'protein supplement' OR 'creatine' OR 'beta alanine' OR 'β-alanine' OR 'leucine' OR 'amino acid' OR 'carbohydrate loading' OR 'branched chain amino acid' OR 'proteins' OR 'protein' OR 'amino acids' OR 'amino' OR 'acids' OR 'whey proteins' OR 'whey protein' OR 'whey' OR 'vitamin' OR 'beetroot juice' OR 'caffeine' OR 'hmb'):ti,ab,kw |
| 5 | MeSH descriptor: [Physical Conditioning, Human] explode all trees |
| 6 | ('Physical Conditioning, Human' OR 'Fitness Training' OR 'Athletic Conditioning' OR 'Strength AND Conditioning' OR 'Sport Training' OR 'Motor Control Training' OR 'Functional Training' OR 'Resistance Training' OR 'Training, Resistance' OR 'Weight Training' OR 'Weight-Lifting Strengthening Program' OR 'Strengthening Program, Weight-Lifting' OR 'Weight Lifting Strengthening Program' OR 'Weight-Lifting Strengthening Programs' OR 'Weight-Lifting Exercise Program' OR 'Exercise Programs, Weight-Lifting' OR 'Exercise Program, Weight-Lifting' OR 'Weight Lifting Exercise Program' OR 'Weight-Lifting Exercise Programs' OR 'Weight-Bearing Strengthening Program' OR 'Strengthening Programs, Weight-Bearing' OR 'Strengthening Program, Weight-Bearing' OR 'Weight Bearing Strengthening Program' OR 'Weight-Bearing Strengthening Programs' OR 'Weight-Bearing Exercise Program' OR 'Exercise Programs, Weight-Bearing' OR 'Exercise Program, Weight-Bearing' OR 'Weight Bearing Exercise Program' OR 'Weight-Bearing Exercise Programs') |
| 7 | ('Plyometric Training' OR 'Plyometric Exercises' OR 'Plyometric Drills' OR 'Exercise, Plyometric' OR 'Exercises, Plyometric' OR 'Training, Plyometric' OR 'Trainings, Plyometric' OR 'Stretch-Shortening Cycle' OR 'Stretch-Shortening Exercises' OR 'Stretch-Shortening Drills' OR 'Stretch-Shortening Exercise' OR 'Stretch-Shortening Cycle Exercise' OR 'Stretch Shortening Exercise' OR 'Stretch Shortening Drill' OR 'Drill, Plyometric' OR 'Drills, Plyometric' OR 'Drill, Stretch-Shortening' OR 'Drills, Stretch-Shortening' OR 'Endurance Training' OR 'Aerobic Training' OR 'Sprint Interval Training' OR 'Sprint Training' OR 'Agility Training' OR 'High-Intensity Interval Training' OR 'HIIT' OR 'Interval Training, High-Intensity' OR 'Interval Trainings, High-Intensity' OR 'Training, High-Intensity Interval' OR 'Exercise, High-Intensity Intermittent' OR 'Exercises, High-Intensity Intermittent' OR 'High-Intensity Intermittent Exercises' OR 'Anaerobic Training' OR 'Anaerobic Exercise' OR 'Anaerobic Capacity' OR 'Sprint Exercise'):ti,ab,kw |
| 8 | MeSH descriptor: [Randomized Controlled Trial] explode all trees |
| 9 | ('randomized controlled trial' OR 'randomized' OR 'clinical trials' OR 'placebo' OR 'randomly' OR 'trial' OR 'rct'):ti,ab,kw |
| 10 | #1 OR #2 |
| 11 | #3 OR #4 |
| 12 | #5 OR #6 OR #7 |
| 13 | #9 OR #9 |
| 14 | #10 AND #11 AND #12 AND #13 |

# Appendix 2: Characteristics of included studies

**Table S2.1:** Baseline of characteristics of included studies

| Author  Year | Country | Group | Sample Size  (M/F) | Age  (Mean ± SD) | Exercise requency Frequency | dietary supplements | | Period | Results |
| --- | --- | --- | --- | --- | --- | --- | --- | --- | --- |
|  |  |  |  |  |  | categories | Exercise requency Frequency |  |  |
| Jones et al. 1999 | Britain | RT | 8/0 | 27.0 ± 4 | 1-2/w | creatine | 20g/d for five days, 5g/d for the following days | 10W | Mean time for 47 m sprint |
|  |  | c | 8/0 | 27.0 ± 4 | 1-2/w | glucose | Same dose |  |  |
| Derave et al. 2007 | Belgium | RT | 8/0 | 18.4 ± 1.5 | 5-6/w | β-alanine | 1–4d: 2.4 g/d,5–8d: 3.6 g/d,Day 9 onward: 4.8 g/d | 6w | 400 m sprint |
|  |  | c | 7/0 | 18.4 ± 1.5 | 5-6/w | maltodextrin | Same dose |  |  |
| Gross et al. 2014 | Switzerland | MMT | 5/0 | 19.5 ± 1.1 | 4-6/w | β-alanine | 4.8g/d | 5w | countermovement jump |
|  |  | C | 4/0 | 19.5 ± 1.1 | 4-6/w | maltodextrin | Same dose |  |  |
| Brisola et al. 2016 | Brazil | RT | 11/0 | 19±5 | 2/d | β-alanine | First 10 days: 4.8 g/day, Last 18 days: 6.4 g/day | 4w | Fastest, mean, and total times of six 10 m sprints |
|  |  | C | 11/0 | 18±3 | 2/d | glucose | Same dose |  |  |
| Fairbairnt et al. 2018a | New Zealand | RT | 28/0 | 21.5 |  | Vitamin D3 | 1.25mg，1/2w | 6w | 10 m sprint, 30 m sprint, lying-pull 1RM, pull-up 1RM, bench-press 1RM |
|  |  | C | 29/0 | 20.9 |  | placebo | Same dose |  |  |
| Fairbairnt et al. 2018b | New Zealand | RT | 28/0 | 21.5 |  | Vitamin D3 | 1.25mg，1/2w | 12w | 10 m sprint, 30 m sprint, lying-pull 1RM, pull-up 1RM, bench-press 1RM |
|  |  | C | 29/0 | 20.9 |  | placebo | Same dose |  |  |
| Guo et al. 2024 | Korea | SSIT | 3/11 | 24.6 ± 2.5 | 3/w | β-alanine | 4.8g/d | 8w | vertical jump, standing long jump, spike jump, block jump, 10 m sprint |
|  |  | C | 4/8 | 23.8 ± 2.7 | 3/w | glucose | Same dose |  |  |
| Pluim et al. 2016 | the Netherlands | CSE | 4/3 | 22.5 ± 4.9 | 3/w | creatine | 6 days: 0.3 g/kg body weight, 6 days later: 0.03 g/kg body weight/d | 6d+4w | Mean & peak bench press, mean & peak overhead press, 5/10/20 m sprints |
|  |  | C | 3/4 | 22.8 ± 4.8 | 3/w | placebo | Same dose |  |  |
| Wu et al. 2024a | China | PT | 9/5 | 21.6 ± 2.5 | 3/w | caffeine | 3 mg/kg/d | 6w | vertical jump |
|  |  | C | 11/10 | 22.8 ± 2.7 | 3/w | placebo | Same dose |  |  |
| Wu et al. 2024b | China | PT | 9/5 | 21.6 ± 2.5 | 3/w | caffeine | 6 mg/kg/d | 6w | vertical jump |
|  |  | C | 11/10 | 22.8 ± 2.7 | 3/w | placebo | Same dose |  |  |
| Menghao et al.2005a | China | SSIT | 8/0 | 20.4 ± 1.2 | 3/w | creatine | 0.1 g·kg/d | 4w | vertical jump, 20 m sprint |
|  |  | SSIT | 8/0 | 20.1 ± 1.6 | 3/w | β-alanine | 4.8 g/d |  |  |
|  |  | C | 8/0 | 20.5 ± 1.4 | 3/w | glucose | Same dose |  |  |
| Menghao et al.2005b | China | SSIT | 8/0 | 20.4 ± 1.2 | 3/w | creatine | 0.1 g·kg/d | 4w | vertical jump, 20 m sprint |
|  |  | SSIT | 8/0 | 20.1 ± 1.6 | 3/w | β-alanine | 4.8 g/d |  |  |
|  |  | C | 8/0 | 20.5 ± 1.4 | 3/w | glucose | Same dose |  |  |
| Jastrzębska et al.2016 | Poland | HIIT | 20/0 | 17.5 ± 0.6 | 4/w | Vitamin D | 5000 IU/d | 8w | 5/10/20 m sprints,squat jump,countermovement jump |
|  |  | C | 16/0 | 17.5 ± 0.6 | 4/w | placebo | Same dose |  |  |
| Stout et al.1999 | America | MMT | 8/0 | 19.6±1.0 | 6/W | creatine+glucose | 5.25g+1g | 8w | Bench-press 1RM, vertical jump, 100-yd sprint, lean body mass |
|  |  | C | 8/0 | 19.6±1.0 | 6/W | glucose | 35g |  |  |

| McIntosh et al. 2018 | New Zealand | RT | 13/0 | 20.3 ± 1.2 | 4/w | HMB | 3g/d | 11w | Bench-press 1RM, squat 1RM, pull-up 1RM, snatch 1RM |
| --- | --- | --- | --- | --- | --- | --- | --- | --- | --- |
|  |  | C | 14/0 | 21.9 ± 2.8 | 4/w | placebo | Same dose |  |  |
| Hoffman et al. 2009a | America | MMT | 13/0 | 19.9±1.3 | 4/w | protein | 42g，Once before and after training | 10w | Bench-press 1RM, squat 1RM, lean body mass |
|  |  | C | 7/0 | 20.7±1.1 | 4/w | placebo | Same dose |  |  |
| Hoffman et al. 2009a | America | MMT | 13/0 | 19.9±1.3 | 4/w | protein | 42g，Once in the morning and once in the evening | 10w | Bench-press 1RM, squat 1RM, lean body mass |
|  |  | C | 7/0 | 20.7±1.1 | 4/w | placebo | Same dose |  |  |
| Slater et al. 2001a | America | RT | 9/0 | 24.9±6.0 | 2-3/W | HMB | 3g/d, (Standard capsules) | 3w | Bench-press 1RM, squat 1RM, leg-press 1RM, total strength |
|  |  | C | 9/0 | 20.4±1.4 | 2-3/W | placebo | Same dose |  |  |
| Slater et al. 2018b | America | RT | 9/0 | 24.9±6.0 | 2-3/W | HMB | 3g/d, (Release Capsule) | 6w | Bench-press 1RM, squat 1RM, leg-press 1RM, total strength |
|  |  | C | 9/0 | 20.4±1.4 | 2-3/W | placebo | Same dose |  |  |
| Slater et al. 2001c | America | RT | 9/0 | 24.9±6.0 | 2-3/W | HMB | 3g/d, (Standard capsules) | 3w | Bench-press 1RM, squat 1RM, leg-press 1RM, total strength |
|  |  | C | 9/0 | 20.4±1.4 | 2-3/W | placebo | Same dose |  |  |
| Slater et al. 2001d | America | RT | 9/0 | 24.9±6.0 | 2-3/W | HMB | 3g/d, (Release Capsule) | 6w | Bench-press 1RM, squat 1RM, leg-press 1RM, total strength |
|  |  | C | 9/0 | 20.4±1.4 | 2-3/W | placebo | Same dose |  |  |
| Jung et al. 2018 | Korea | MMT | 12/18 | 20.1±0.15 | 5/w | Vitamin D₃ | 5000 IU/d | 4/w | countermovement jump |
|  |  | C | 9/6 | 20.1±0.15 | 5/w | placebo | Same dose |  |  |
| Lis et al. 2022a | America | MMT | 24/0 | 18.8±2.0 | 3/w | protein | 20g+50mg | 2w | countermovement jump |
|  |  | C | 24/0 | 18.8±2.0 | 3/w | maltodextrin | 20g |  |  |
| Lis et al. 2022a | America | MMT | 24/0 | 18.8±2.0 | 3/w | protein | 20g+50mg | 3w | countermovement jump |
|  |  | C | 24/0 | 18.8±2.0 | 3/w | maltodextrin | 20g |  |  |
| Percario et al.2012 | Brazil | RT | 9/0 | 17.1±1.63 | 3-4/W | creatine | In the first 5 days, 20g/d; After 5 days, 5g/d | 32d | Bench-press 1RM, muscle mass |
|  |  | C | 9/0 | 17.1±1.63 | 3-4/W | maltodextrin | Same dose |  |  |
| Haff et al. 2000 | America | MMT | 7/8 | 19.9±0.4 | 5/w | creatine | 0.3 g/kg/d | 6w | countermovement jump,squat jump,lean body mass |
|  |  | C | 9/12 | 19.9±0.4 | 5/w | maltodextrin | Same dose |  |  |
| Wilder et al. 2002a | America | RT | 8/0 | 18.8±0.99 | 4/w | creatine | 3g//d | 5w | Squat 1RM, lean body mass |
|  |  | C | 9/0 | 19.2±1.09 | 4/w | glucose | Same dose |  |  |
| Wilder et al. 2002b | America | RT | 8/0 | 18.8±0.99 | 4/w | creatine | 3g//d | 10w | Squat 1RM, lean body mass |
|  |  | C | 9/0 | 19.2±1.09 | 4/w | glucose | Same dose |  |  |
| Wilder et al. 2002c | America | RT | 8/0 | 18.8±0.99 | 4/w | creatine | 1w：20 g/d；2–10w：5 g/d | 5w | Squat 1RM, lean body mass |
|  |  | C | 9/0 | 19.2±1.09 | 4/w | glucose | Same dose |  |  |
| Landa et al. 2020 | Spain | RT | 7/0 | 30.43±4.65 | 6/w | HMB | 0.04 g·kg/d | 10w | lean body mass |
|  |  | C | 7/0 | 30.43±4.65 | 6/w | placebo | Same dose |  |  |
| Bezrati et al. 2020 | Spain | MMT | 19/0 | 10.7±2.15 | 1/w | Vitamin D | 200,000 IU/d | 12w | vertical jump,standing long jump,10m/20msprint |
|  |  | C | 17/0 | 10.8±12.2 | 1/w | placebo | Same dose |  |  |

| Wilder et al. 2002d | America | RT | 8/0 | 18.8±0.99 | 4/w | creatine | 1w：20 g/d；2–10w：5 g/d | 10w | Squat 1RM, lean body mass |
| --- | --- | --- | --- | --- | --- | --- | --- | --- | --- |
|  |  | C | 9/0 | 19.2±1.09 | 4/w | glucose | Same dose |  |  |
| Bemben et al. 2001a | America | MMT | 9/0 | 19.4±0.1 | 4/w | creatine | .1–5d：20 g/d,6d-9w：5 g/d | 9w | Bench-press 1RM, squat 1RM, snatch 1RM, lean body mass |
|  |  | C | 8/0 | 19.3±0.5 | 4/w | glucose | Same dose |  |  |
| Bemben et al. 2001b | America | MMT | 9/0 | 19.4±0.1 | 4/w | creatine | .1–5d：20 g/d,6d-9w：5 g/d | 9w | Bench-press 1RM, squat 1RM, snatch 1RM, lean body mass |
|  |  | C | 8/0 | 19.0±0.3 | 4/w | placebo | Same dose |  |  |
| Hoffman et al. 2007 | America | RT | 11/0 | 20.3±1.6 | 4/w | protein | 84/d | 10w | Bench-press 1RM, squat 1RM, lean body mass |
|  |  | C | 10/0 | 21.0±1.2 | 4/w | maltodextrin | Same dose |  |  |
| Pearson et al. 1999 | America | RT | 8/0 | 20.7 | 3/w | creatine | 5g/d | 10w | Bench-press 1RM, squat 1RM, snatch 1RM |
|  |  | C | 8/0 | 20.7 | 3/w | placebo | Same dose |  |  |
| Portal et al. 2011 | Israel | MMT | 7/7 | 16.1±1.3 | 4-5/w | HMB | 3g/d | 7w | Bench-press 1RM, leg-press 1RM |
|  |  | C | 7/7 | 16.2±1.3 | 4-5/w | placebo | Same dose |  |  |
| Wang et al. 2025 | China | RT | 12/0 | 21±1 | 2/w | caffeine | 3g·kg/d | 4w | countermovement jump,vertical jump |
|  |  | C | 12/0 | 21±1 | 2/w | placebo | Same dose |  |  |
| Taylor et al. 2016 | America | MMT | 0/8 | 20±2 | 4/w | protein | 24g/d  /g | 8w | Bench-press 1RM, leg-press 1RM, vertical jump, standing long jump (standing long jump), lean body mass |
|  |  | C | 0/6 | 20±3 | 4/w | maltodextrin | Same dose |  |  |
| Obradović et al. 2020 | Serbia | RT | 10/0 | 23.92±1.54 | 4/w | protein | 20g/d | 8w | Bench-press 1RM, squat 1RM, shoulder-press 1RM, lean body mass, muscle mass |
|  |  | C | 10/0 | 23.92±1.54 | 4/w | maltodextrin | 10g/d |  |  |
| Rosas et al. 2017 | Argentina | PT | 0/8 | 24.3±2.5 | 2/w | β-alanine | 2.4g/d | 6w | squat jump,countermovement jump,20m sprint |
|  |  | C | 0/8 | 22.8±2.1 | 2/w | placebo | Same dose |  |  |
| Molina et al. 2022 | Spain | MMT | 12/0 | 14.3±0.5 | 4/w | creatine | 0.1 g·kg/d | 8w | squat jump,DJ,Abalakov jump |
|  |  | C | 12/0 | 14.4±0.5 | 4/w | placebo | Same dose |  |  |
| Campillo et al.2016 | Chile | PT | 0/10 | 23.1±3.4 | 2/w | creatine | 1w:20g/d,2-6w:5g/d | 6/w | squat jump，countermovement jump,RAST Mean Sprint Time,20m sprint |
|  |  | C | 0/10 | 22.9±1.7 | 2/w | glucose | Same dose |  |  |
| Wang et al. 2018 | ChinaTaiwan | MMT | 15/0 | 20±2 | 3/w | creatine | 1w:20g/d,2-4w:2g/d | 4w | 30 m sprint, half-squat 1RM, lean body mass, vertical jump |
|  |  | C | 15/0 | 20±1 | 3/w | placebo | Same dose |  |  |
| Burke et al. 2000 | Canada | RT | 10/0 | 21±2 | 3/w | creatine | 5g/d | 6w | lean body mass |
|  |  | C | 10/0 | 21±1 | 3/w | maltodextrin | Same dose |  |  |
| Huang et al. 2017 | ChinaTaiwan | MMT | 6/0 | 21.7±2.7 |  | protein | 33.5/g | 5w | lean body mass |
|  |  | C | 6/0 | 21.7±2.0 |  | maltodextrin | Same dose |  |  |
| Kreiderr et al. 2000 | America | RT | 16/0 | 20.0±1.5 | 7/w | HMB | 3g/d | 28d | lean body mass |
|  |  | C | 19/0 | 20.0±1.5 | 7/w | placebo | Same dose |  |  |

Note:RT,Resistance Training;PT,Plyometric;SSIT,Short-Sprint Interval;HIIT,High-Intensity Interval Training;CSE,Concurrent Strength + Endurance;MMTMulti-Modal Training.

# Appendix 3: Risk of bias of randomized clinical trials

**Figure 2:** Overall risk of bias presented as percentage of each risk of bias item across all included studies. Green = Low risk, Red = High risk, Yellow = Some concerns.


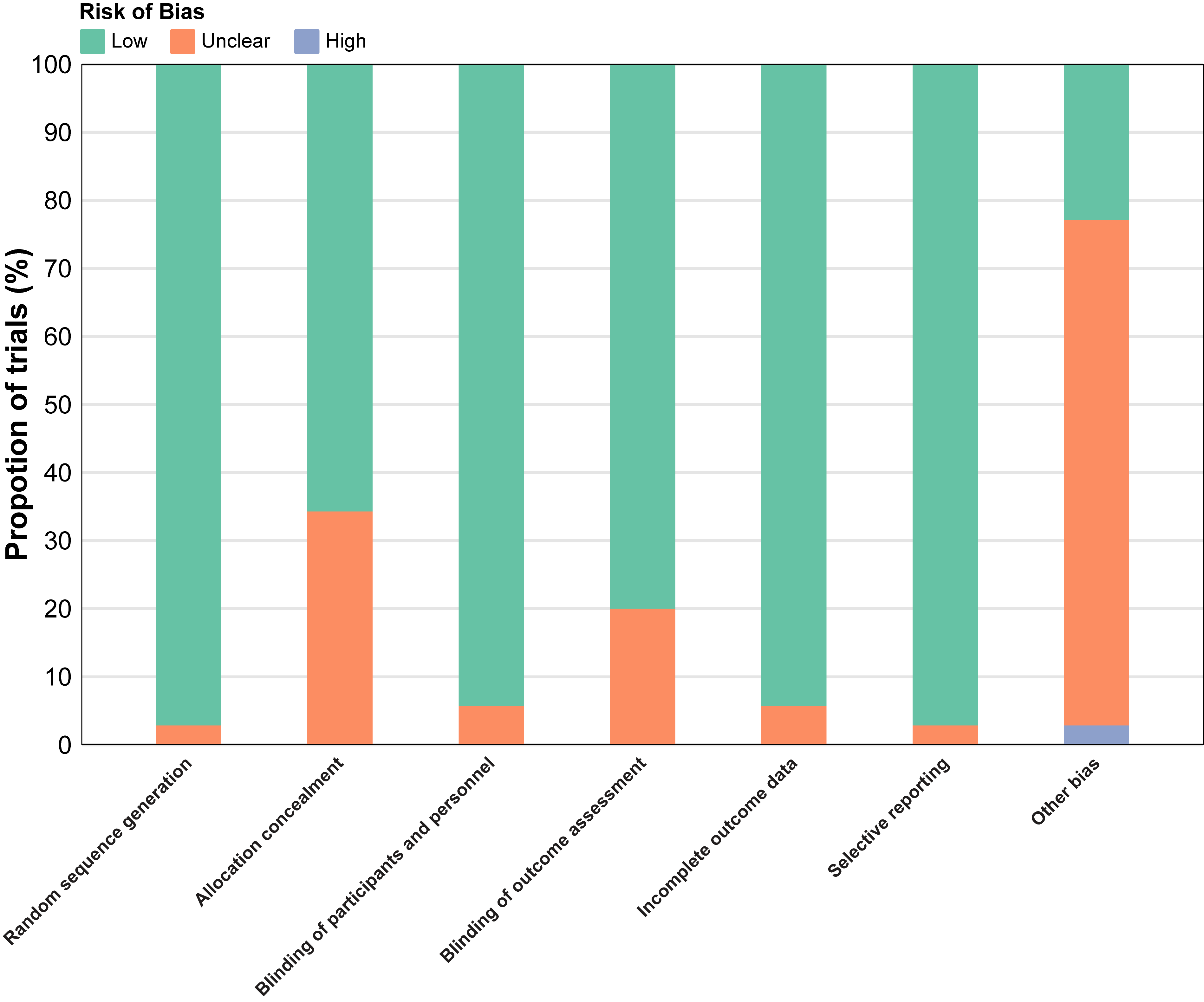


**Figure S2:** Study level risk of bias assessment using Cochrane risk of bias tool 2.0 for assessing risk of bias of randomized clinical trials.


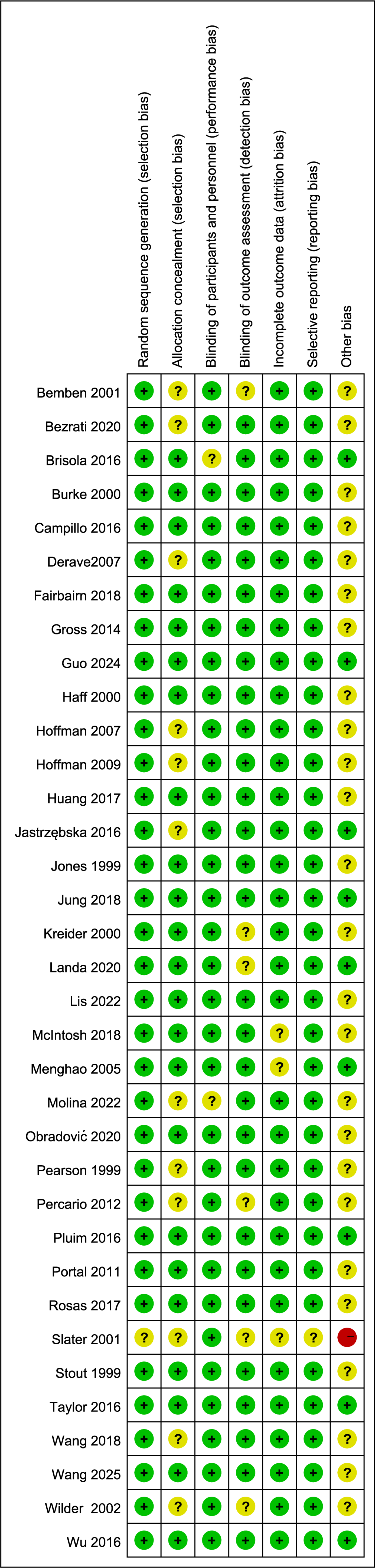


# Appendix 4: Evaluation of inconsistency and heterogeneity

**Table S4.1** Network Heterogeneity Assessment Results of muscle Sstrength

| τ² | τ | I² (%) |
| --- | --- | --- |
| 0.0947 | 0.3077 | 35.5% |

**Table S4.2** Network Heterogeneity Assessment Results of jumping performance

| τ² | τ | I² (%) |
| --- | --- | --- |
| 0 | 0 | 0% |

**Table S4.3** Results of Inconsistencies in Node Segmentation of jumping performance

| comparison | k | prop | prop | direct | indir. | Diff | z | p-value |
| --- | --- | --- | --- | --- | --- | --- | --- | --- |
| creatine:placebo | 12 | 0.97 | 0.3003 | 0.3027 | 0.2204 | 0.0823 | 0.12 | 0.9073 |
| creatine:β-alanine | 2 | 0.28 | -0.1094 | 0.0000 | -0.1528 | 0.1528 | 0.37 | 0.7147 |
| placebo:β-alanine | 9 | 0.94 | -0.4097 | -0.4385 | 0.0356 | -0.4741 | -0.71 | 0.4771 |

**Table S4.4** Network Heterogeneity Assessment Results of sprinting speed

| τ² | τ | I² (%) |
| --- | --- | --- |
| 0 | 0 | 0% |

**Table S4.5** Results of Inconsistencies in Node Segmentation of sprinting speed

| comparison | k | prop | prop | direct | indir. | Diff | z | p-value |
| --- | --- | --- | --- | --- | --- | --- | --- | --- |
| placebo:creatine | 10 | 0.96 | 0.4224 | 0.4424 | -0.1051 | 0.5475 | 0.78 | 0.4339 |
| β-alanine:creatine | 2 | 0.29 | 0.1876 | 0.0924 | 0.2264 | -0.1340 | -0.32 | 0.7494 |
| placebo:β-alanine | 9 | 0.95 | 0.2347 | 0.2426 | 0.0957 | 0.1469 | 0.22 | 0.8277 |

**Table S4.6** Network Heterogeneity Assessment Results of muscle mass

| τ² | τ | I² (%) |
| --- | --- | --- |
| 0 | 0 | 0% |

# Appendix 5: Network maps and forest plots of secondary outcomes

Figure S5.1: Network map of the effect on muscle strength, and forest plot of network effect sizes for compared with control. The size of the nodes was proportional to the number of participants included in the trial, and the thickness of lines between the interventions relates to the number of studies for that comparison.


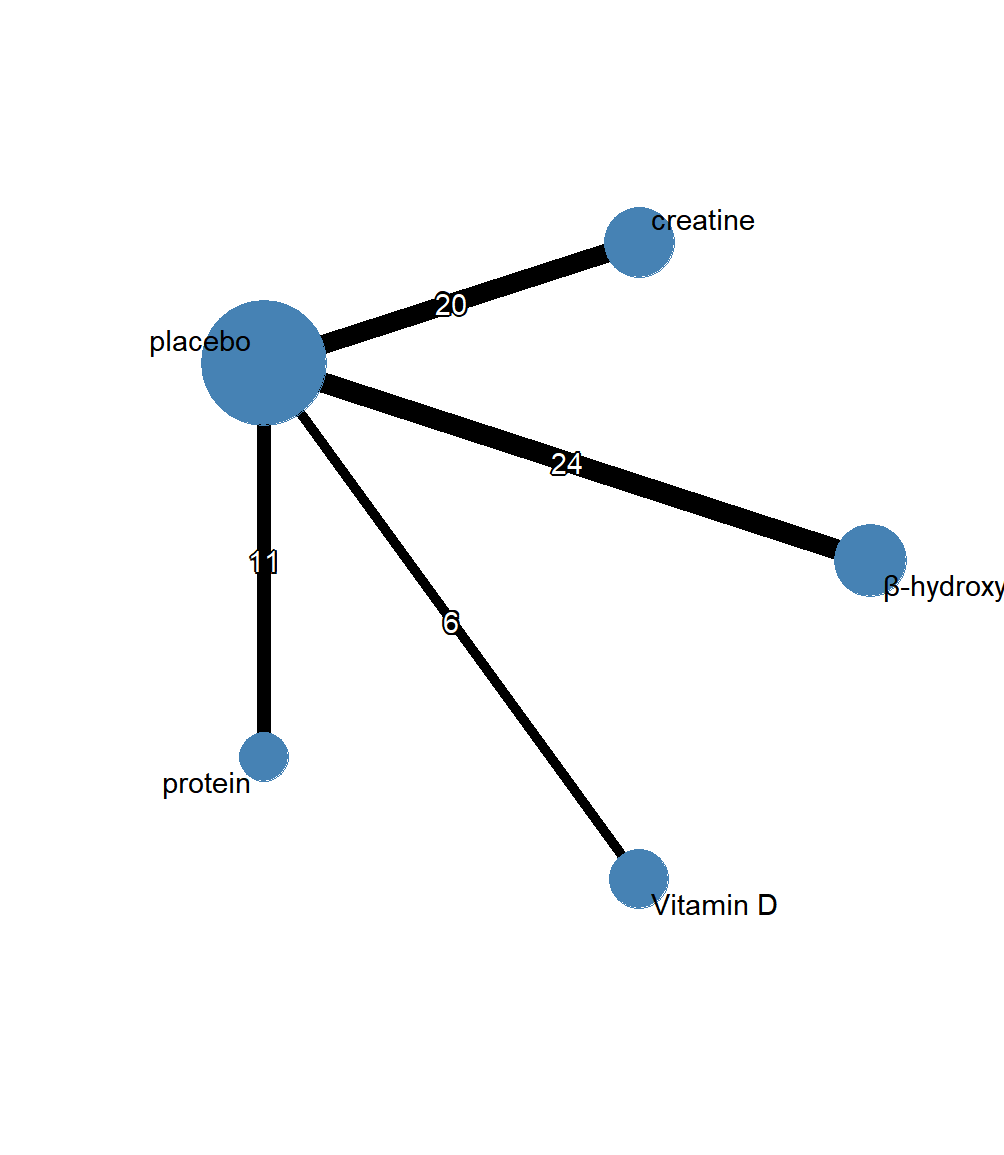


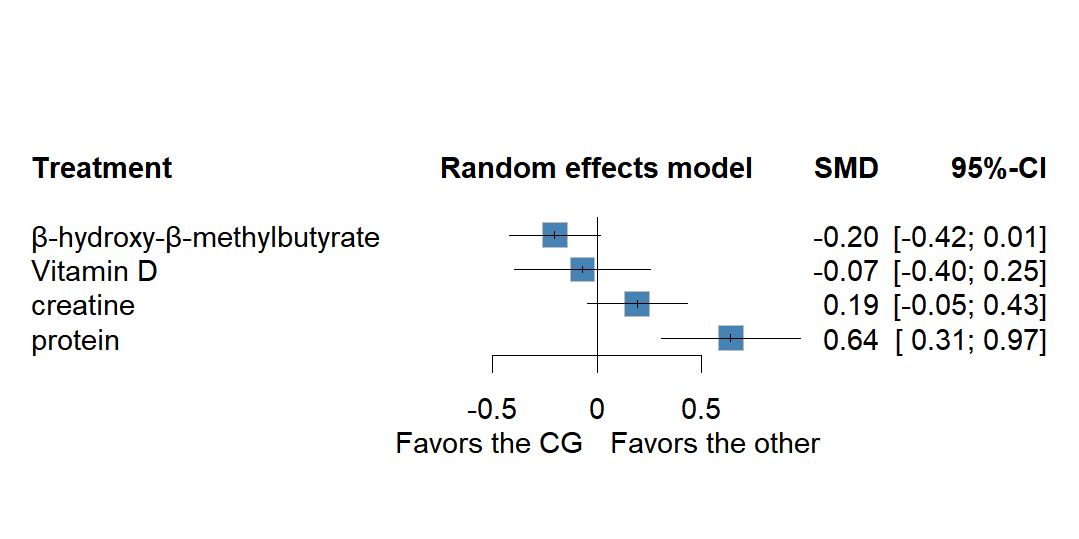


**Figure S5.2:** Network map of the effect on jumping performance, and forest plot of network effect sizes for compared with control. The size of the nodes was proportional to the number of participants included in the trial, and the thickness of lines between the interventions relates to the number of studies for that comparison.


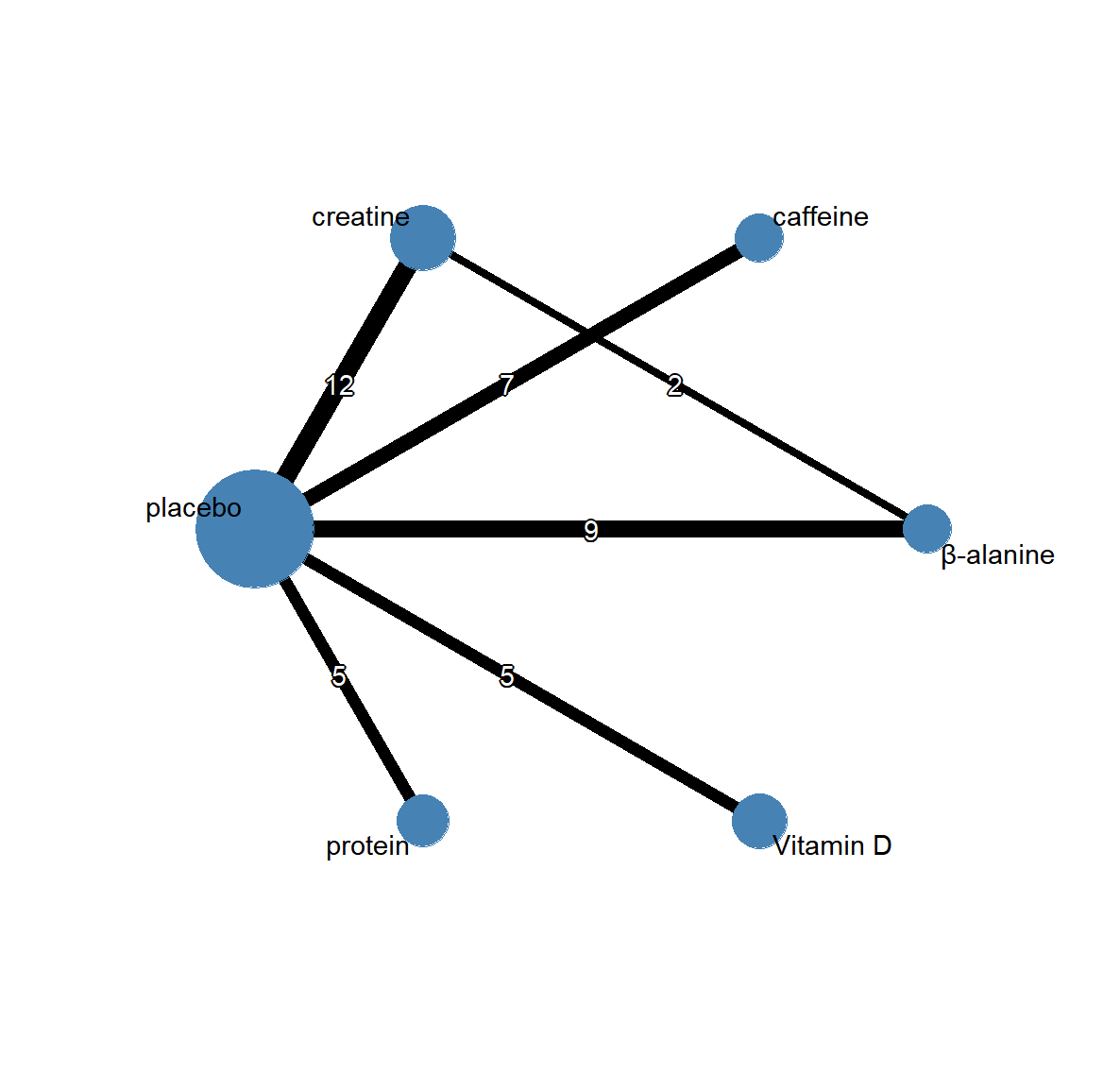


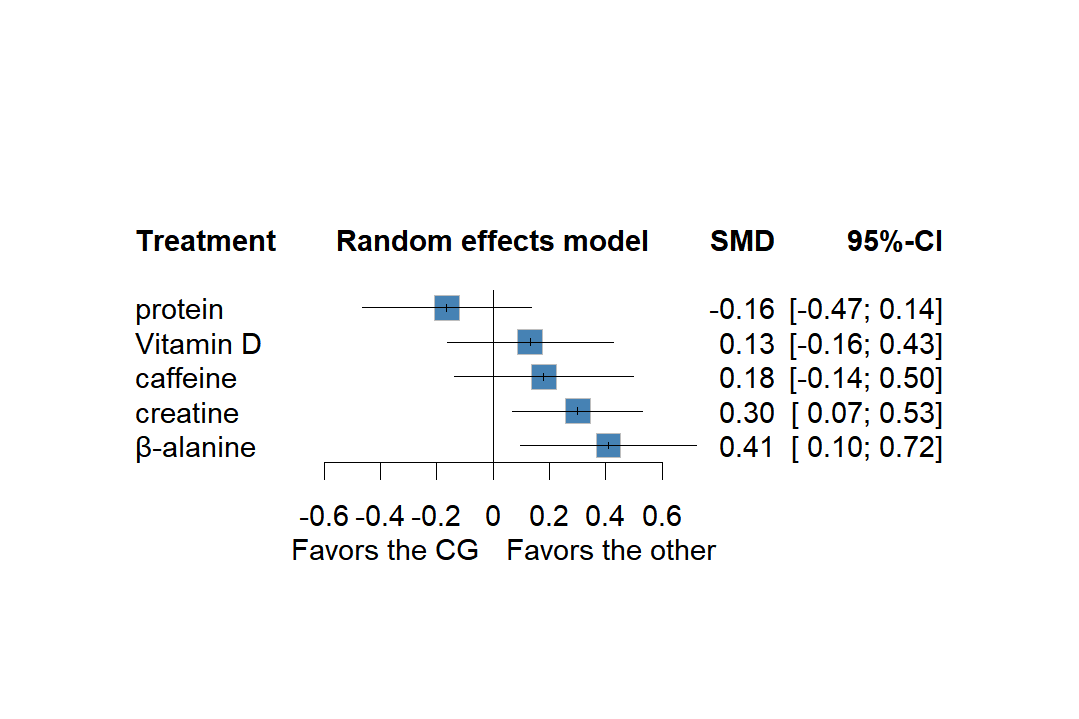


**Figure S5.3:** Network map of the effect on sprinting speed, and forest plot of network effect sizes for compared with control. The size of the nodes was proportional to the number of participants included in the trial, and the thickness of lines between the interventions relates to the number of studies for that comparison.


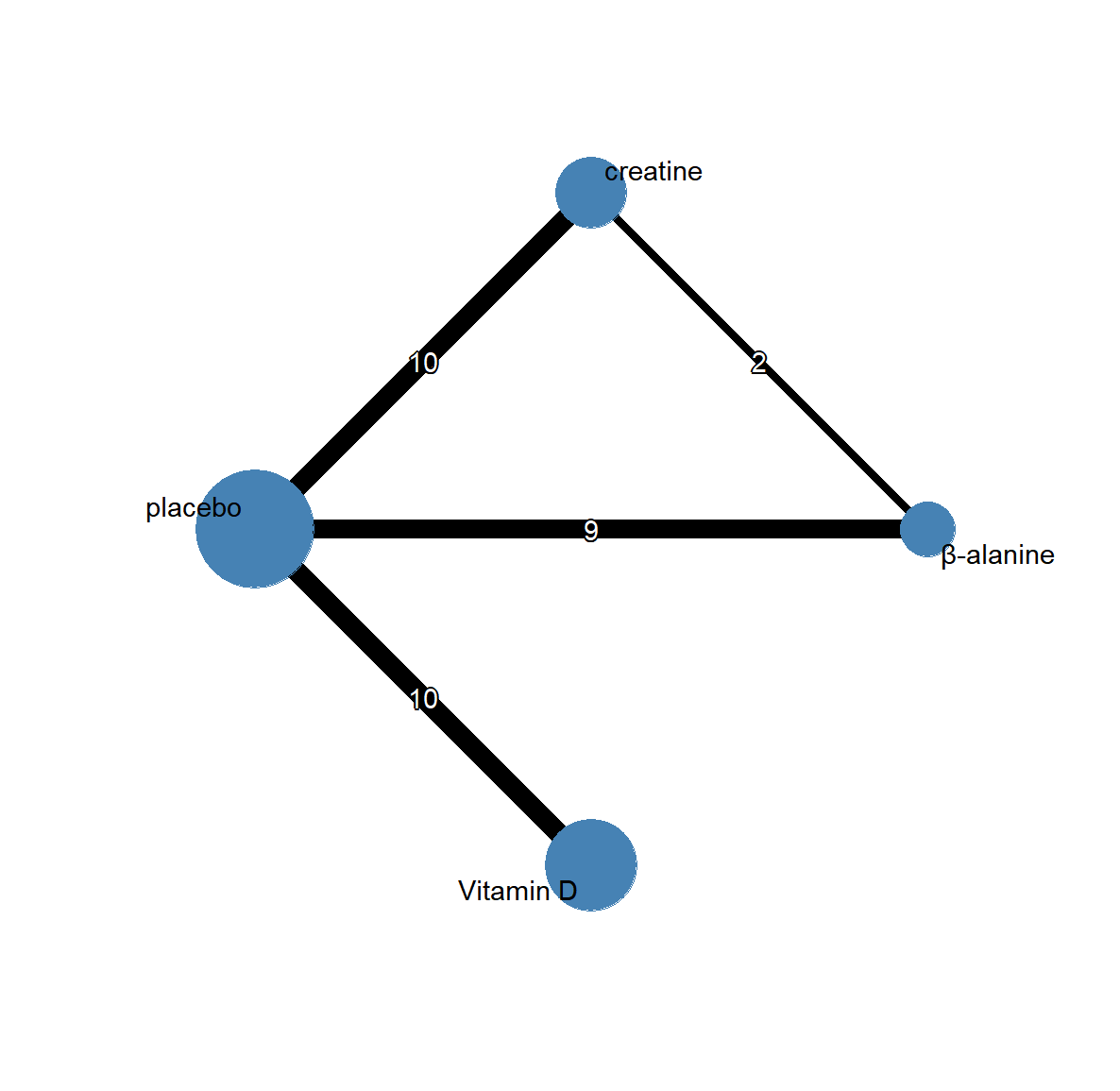


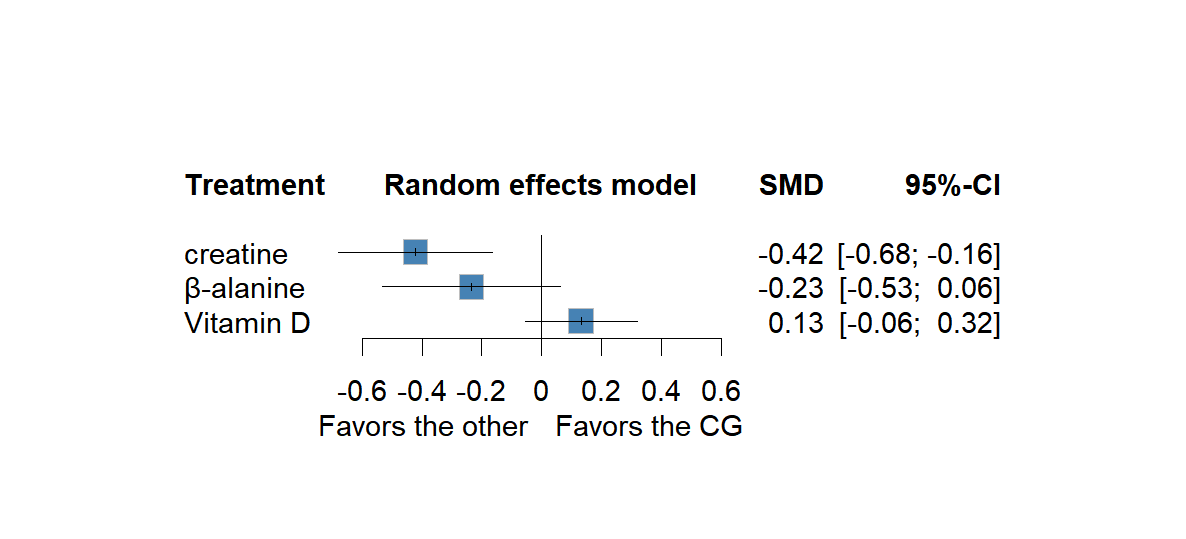


**Figure S5.4:** Network map of the effect on muscle mass, and forest plot of network effect sizes for compared with control. The size of the nodes was proportional to the number of participants included in the trial, and the thickness of lines between the interventions relates to the number of studies for that comparison.


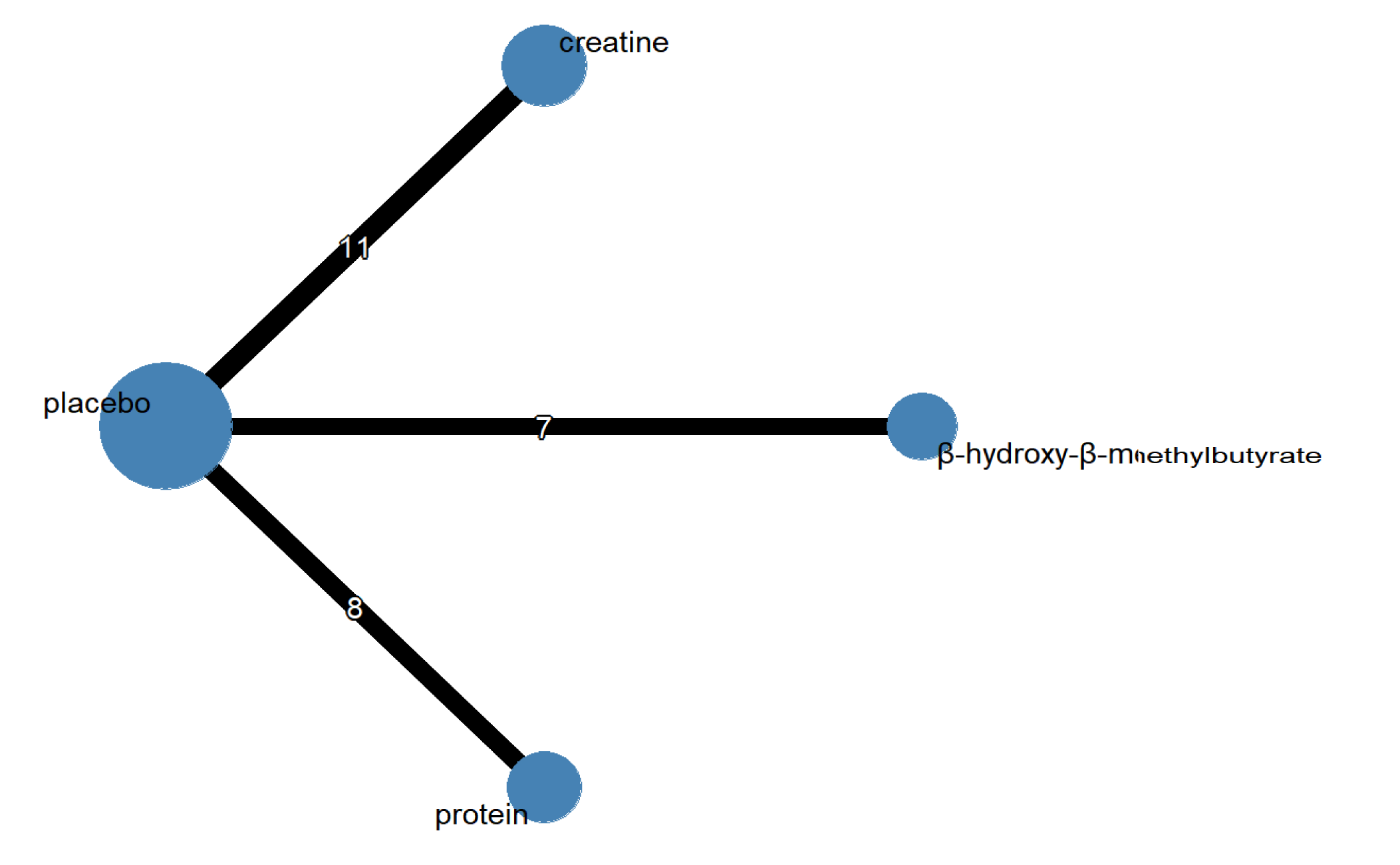


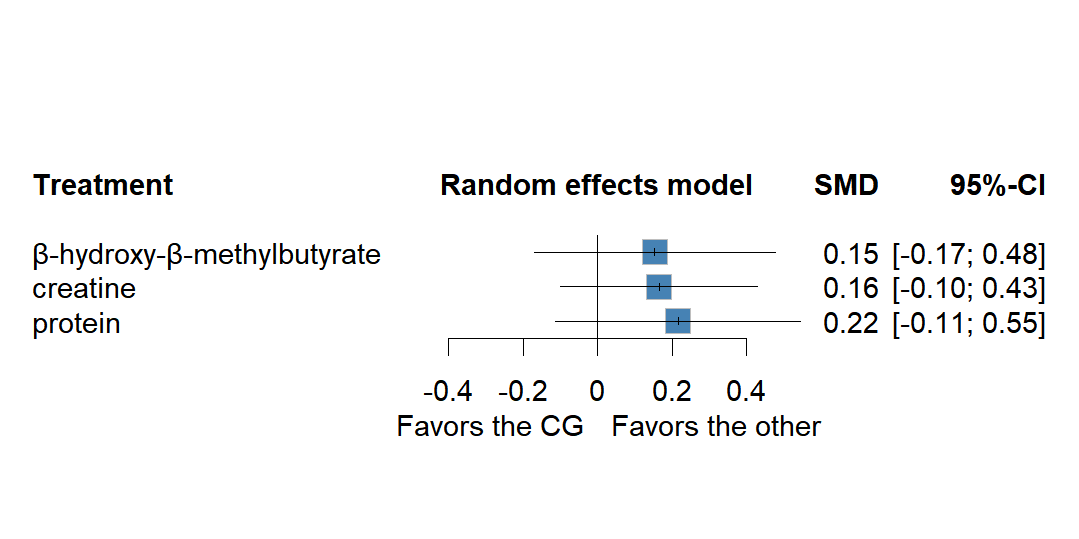


# Appendix 6: SUCRA and cumulative probability plots

**Figure S6.1:** The rank heat plot presents a summary of P scores (range 0-100) for each intervention across outcomes, where darker shades of green represent more benefit and darker shades of red represent less benefit. Beta-alanine=β-alanine; HMB=β-hydroxy-β-methylbutyrate.

**Table S6.1:** SUCRA of the effects of different exercise modality on muscle strength.

| **Treatment** | **SUCRA** |
| --- | --- |
| protein | 0.9956 |
| creatine | 0.7121 |
| placebo | 0.4225 |
| Vitamin D | 0.2956 |
| HMB | 0.0742 |

**Table S6.1:** SUCRA of the effects of different exercise modality on jumping performance

| **Treatment** | **SUCRA** |
| --- | --- |
| β-alanine | 0.8900 |
| creatine | 0.7606 |
| caffeine | 0.5643 |
| Vitamin D | 0.4858 |
| placebo | 0.2388 |
| protein | 0.0605 |

**Table S6.1:** SUCRA of the effects of different exercise modality on sprinting speed

| **Treatment** | **SUCRA** |
| --- | --- |
| creatine | 0.9457 |
| β-alanine | 0.6931 |
| placebo | 0.3261 |
| Vitamin D | 0.0350 |

**Table S6.1:** SUCRA of the effects of different exercise modality on muscle mass

| **Treatment** | **SUCRA** |
| --- | --- |
| protein | 0.6991 |
| creatine | 0.6052 |
| HMB | 0.5667 |
| placebo | 0.1290 |

# Appendix 7: League Table of Summary Estimates for Dietary Supplementation Combined with Strength and Conditioning on Athletic Performance from on Network Meta-Analysis

**Table S7.1**: league table of muscle strength

The columns represent the comparison of the row exercise modality to the column exercise modality. The rows represent the comparison of the row exercise modality to the column exercise modality. The effect estimates are expressed as mean difference and 95% confidence interval. Mean difference <0 favors the exercise modality in the column, and mean difference >0 favors the exercise modality in the row.

| creatine | . | . | . |  |
| --- | --- | --- | --- | --- |
| -0.43 (-0.82; -0.04) | protein | . | . |  |
| 0.33 ( 0.02; 0.64) | 0.76 ( 0.38; 1.13) | HMB | . |  |
| 0.26 (-0.12; 0.63) | 0.69 ( 0.25; 1.12) | -0.07 (-0.43; 0.30) | Vitamin D |  |
| 0.19 (-0.04; 0.42) | 0.61 ( 0.30; 0.93) | -0.14 (-0.35; 0.07) | -0.07 (-0.37; 0.23) | placebo |

**Table S7.2**: league table of jumping performance

The columns represent the comparison of the row exercise modality to the column exercise modality. The rows represent the comparison of the row exercise modality to the column exercise modality. The effect estimates are expressed as mean difference and 95% confidence interval. Mean difference <0 favors the exercise modality in the column, and mean difference >0 favors the exercise modality in the row.

| creatine | . |  | . | . |  |
| --- | --- | --- | --- | --- | --- |
| 0.46 ( 0.08; 0.84) | protein | . | . | . |  |
| -0.11 (-0.48; 0.26) | -0.57 (-1.01; -0.14) | β-alanine | . | . |  |
| 0.12 (-0.27; 0.51) | -0.34 (-0.78; 0.09) | 0.23 (-0.22; 0.68) | caffeine | . |  |
| 0.17 (-0.21; 0.54) | -0.30 (-0.72; 0.13) | 0.28 (-0.15; 0.71) | 0.05 (-0.39; 0.48) | Vitamin D |  |
| 0.30 ( 0.07; 0.53) | -0.16 (-0.47; 0.14) | 0.41 ( 0.10; 0.72) | 0.18 (-0.14; 0.50) | 0.13 (-0.16; 0.43) | placebo |

**Table S7.3**: league table of sprinting speed

The columns represent the comparison of the row exercise modality to the column exercise modality. The rows represent the comparison of the row exercise modality to the column exercise modality. The effect estimates are expressed as mean difference and 95% confidence interval. Mean difference <0 favors the exercise modality in the column, and mean difference >0 favors the exercise modality in the row.

| creatine |  | . |  |
| --- | --- | --- | --- |
| -0.19 (-0.56; 0.19) | β-alanine | . |  |
| -0.55 (-0.87; -0.24) | -0.37 (-0.72; -0.01) | Vitamin D |  |
| -0.42 (-0.68; -0.16) | -0.23 (-0.53; 0.06) | 0.13 (-0.06; 0.32) | placebo |

**Table S7.3**: league table of muscle mass

The columns represent the comparison of the row exercise modality to the column exercise modality. The rows represent the comparison of the row exercise modality to the column exercise modality. The effect estimates are expressed as mean difference and 95% confidence interval. Mean difference <0 favors the exercise modality in the column, and mean difference >0 favors the exercise modality in the row.

| creatine | . | . |  |
| --- | --- | --- | --- |
| -0.05 (-0.47; 0.37) | protein | . |  |
| 0.01 (-0.41; 0.43) | 0.06 (-0.40; 0.52) | HMB |  |
| 0.16 (-0.10; 0.43) | 0.22 (-0.11; 0.55) | 0.15 (-0.17; 0.48) | placebo |

# Appendix 8: CINeMA Assessment

We use the CINeMA framework to assess evidence certainty, evaluating each network estimate based on the following criteria:

- **Within study bias:** We classified the overall risk of bias for each study as low risk of bias, the risk of bias as moderate when none of the four assessed risk of bias items were rated as high risk, and the risk of bias as high when one or both items were rated as high risk. See **Appendix 3** for the bias assessment.
- **Reporting bias:** We judged it visually by a funnel plot **(Appendix 9)**.
- **Indirectness:** Indirectness was judged low when all included studies directly matched our predefined PICO framework in terms of patient population, interventions, comparators, and outcome measurements, with no substantive deviations from the protocol.
- **Imprecision:** We use the CINeMA website to grade the accuracy of each comparison.
- **Heterogeneity:** We assessed the degree of worry by comparing clinical reasoning based on 95% confidence intervals (CIs) while applying the same clinical reasoning framework as for inaccuracy. In particular, we judged the consistency of our findings based on the confidence and prediction intervals associated with clinically important effect sizes. And we used the same thresholds of clinical significance as described above and followed the recommendations automatically provided by CINeMA (https://cinema.ispm.unibe.ch/).
- **Inconsistency:** For inconsistency, we looked at the results for node splitting (Appendix 4).

**Figure S8.1:** Risk of bias contribution by intervention group in muscle strength


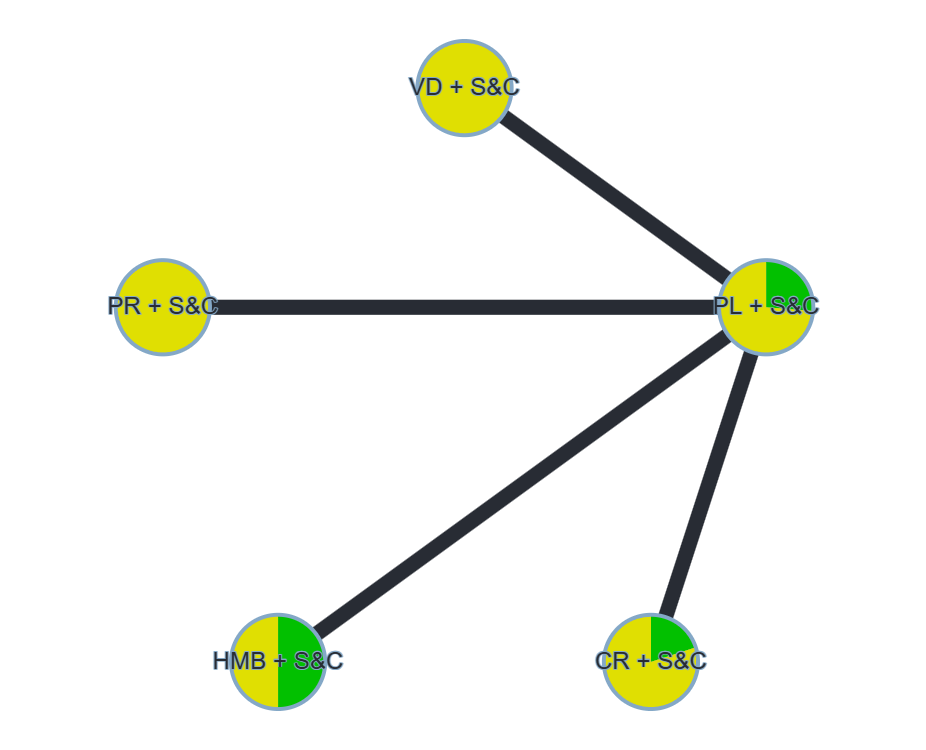


1

**Figure S8.2:** Overall risk of bias by treatment comparison in muscle strength


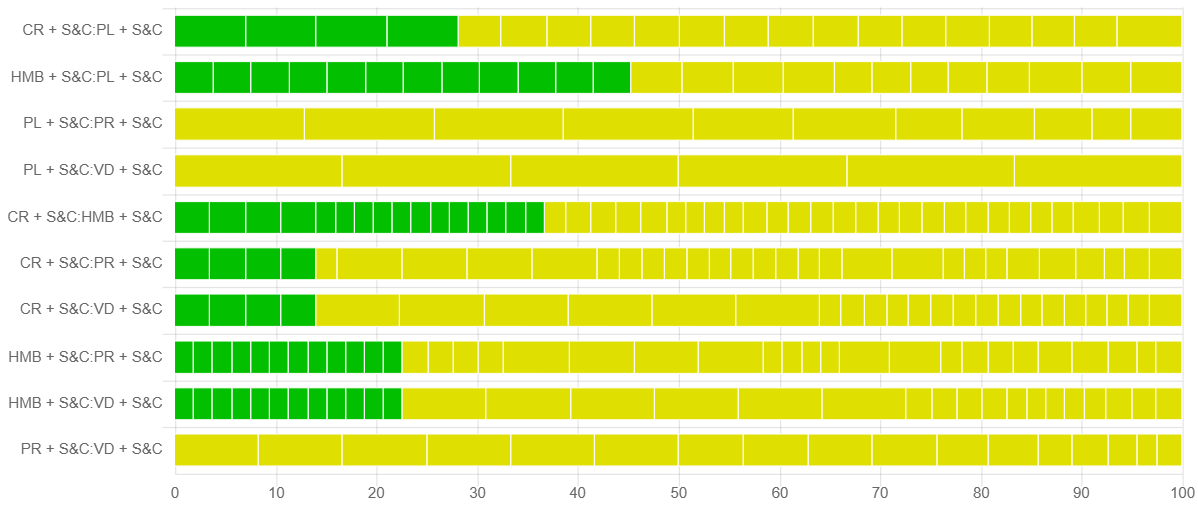


# Figure S8.3: Risk of bias contribution by intervention group in jumping performance


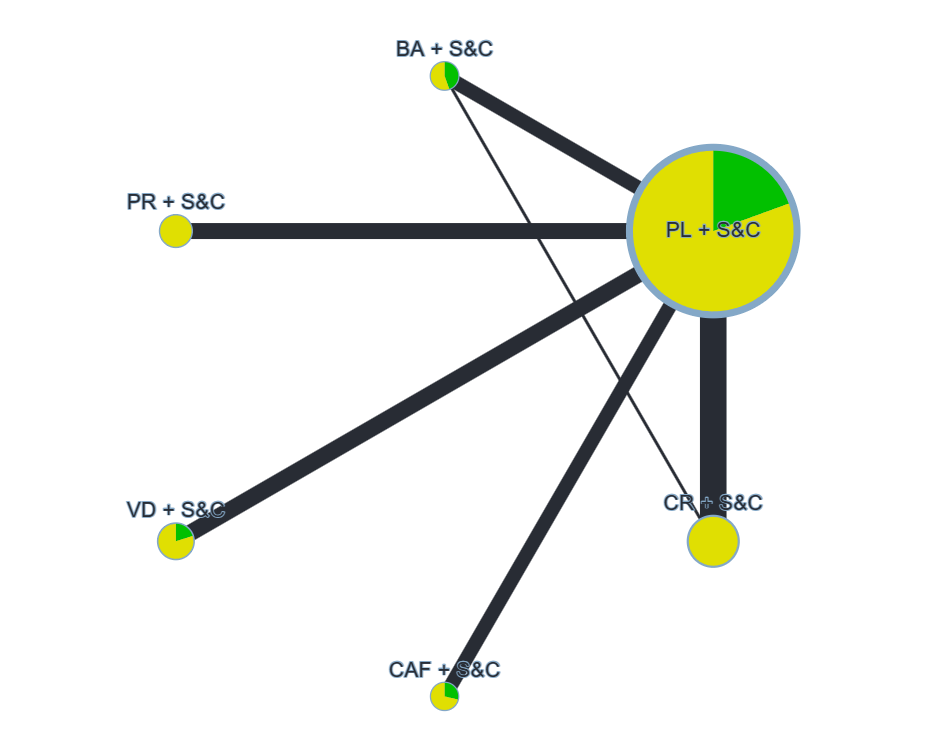


**Figure S8.4:** Overall risk of bias by treatment comparison in jumping performance


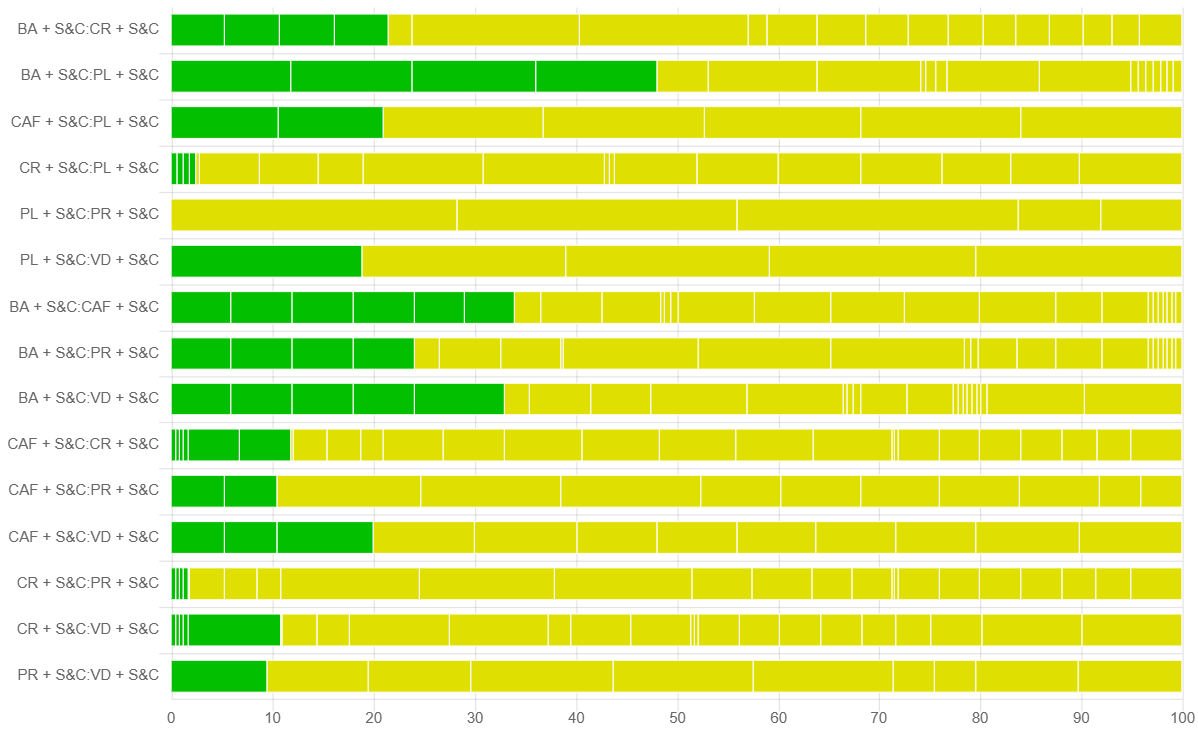


**Figure S8.5**: Risk of bias contribution by intervention group in sprinting speed


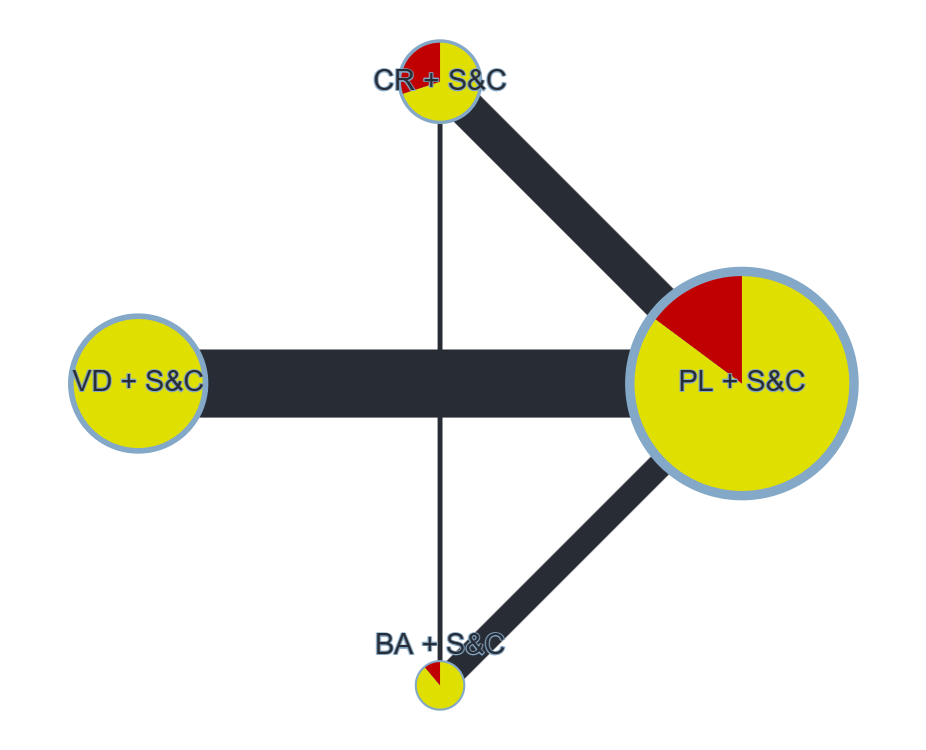


**Figure S8.6:** Overall risk of bias by treatment comparison in sprinting speed


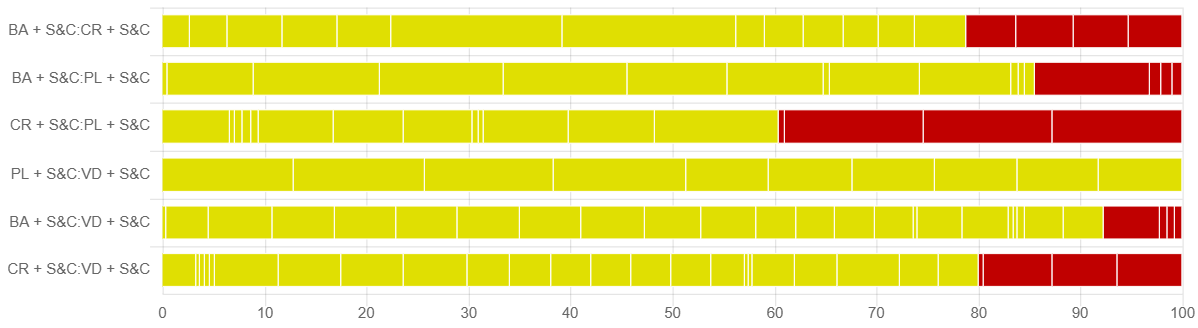


**Figure S8.7:** Risk of bias contribution by intervention group in muscle mass


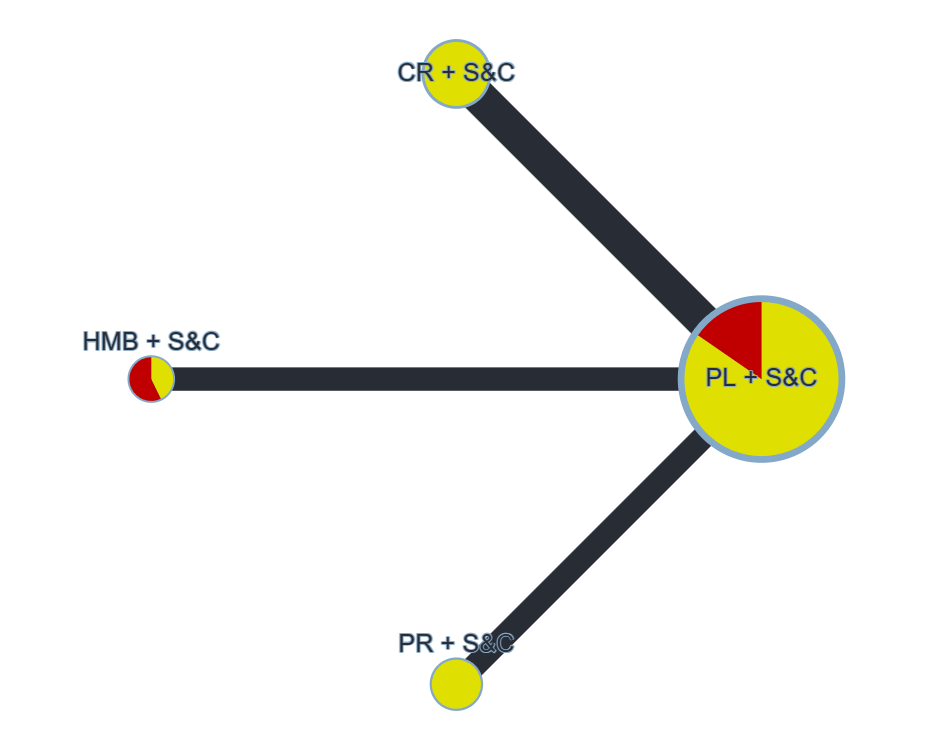


**Figure S8.8:** Overall risk of bias by treatment comparison in muscle mass


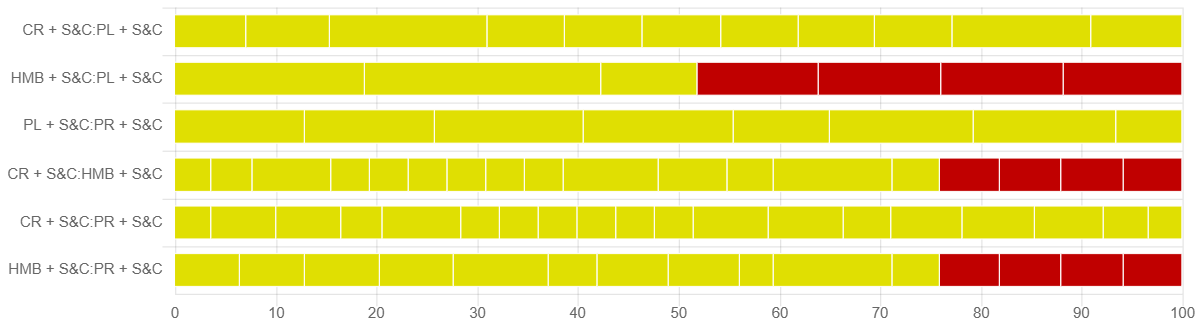


**Table S8.1:** CINeMA Results of muscle strength

| Comparison | Within-study bias | Reporting bias | Indirectness | Imprecision | Heterogeneity | Incoherence | Confidence rating |
| --- | --- | --- | --- | --- | --- | --- | --- |
| CR + S&C:PL + S&C | Some concerns | Low risk | No concerns | Major concerns | No concerns | Major concerns | Very low |
| HMB + S&C:PL + S&C | Some concerns | Low risk | No concerns | Major concerns | No concerns | Major concerns | Very low |
| PL + S&C:PR + S&C | Some concerns | Low risk | No concerns | No concerns | No concerns | Major concerns | Very low |
| PL + S&C:VD + S&C | Some concerns | Low risk | No concerns | Major concerns | No concerns | Major concerns | Very low |
| CR + S&C:HMB + S&C | Some concerns | Low risk | No concerns | No concerns | Major concerns | Major concerns | Very low |
| CR + S&C:PR + S&C | Some concerns | Low risk | No concerns | Major concerns | No concerns | Major concerns | Very low |
| CR + S&C:VD + S&C | Some concerns | Low risk | No concerns | Major concerns | No concerns | Major concerns | Very low |
| HMB + S&C:PR + S&C | Some concerns | Low risk | No concerns | No concerns | No concerns | Major concerns | Very low |
| HMB + S&C:VD + S&C | Some concerns | Low risk | No concerns | Major concerns | No concerns | Major concerns | Very low |
| PR + S&C:VD + S&C | Some concerns | Low risk | No concerns | No concerns | Major concerns | Major concerns | Very low |

**Table S8.2:** CINeMA Results of jumping performance

| Comparison | Within-study bias | Reporting bias | Indirectness | Imprecision | Heterogeneity | Incoherence | Confidence rating |
| --- | --- | --- | --- | --- | --- | --- | --- |
| BA + S&C:CR + S&C | Some concerns | Low risk | No concerns | Major concerns | No concerns | No concerns | Very low |
| BA + S&C:PL + S&C | Some concerns | Low risk | No concerns | No concerns | No concerns | No concerns | Moderate |
| CAF + S&C:PL + S&C | Some concerns | Low risk | No concerns | Major concerns | No concerns | No concerns | Very low |
| CR + S&C:PL + S&C | Some concerns | Low risk | No concerns | No concerns | No concerns | No concerns | Moderate |
| PL + S&C:PR + S&C | Some concerns | Low risk | No concerns | Major concerns | No concerns | No concerns | Very low |
| PL + S&C:VD + S&C | Some concerns | Low risk | No concerns | Major concerns | No concerns | No concerns | Very low |
| BA + S&C:CAF + S&C | Some concerns | Low risk | No concerns | Major concerns | No concerns | No concerns | Very low |
| BA + S&C:PR + S&C | Some concerns | Low risk | No concerns | No concerns | No concerns | No concerns | Moderate |
| BA + S&C:VD + S&C | Some concerns | Low risk | No concerns | Major concerns | No concerns | No concerns | Very low |
| CAF + S&C:CR + S&C | Some concerns | Low risk | No concerns | Major concerns | No concerns | No concerns | Very low |
| CAF + S&C:PR + S&C | Some concerns | Low risk | No concerns | Major concerns | No concerns | No concerns | Very low |
| CAF + S&C:VD + S&C | Some concerns | Low risk | No concerns | Major concerns | No concerns | No concerns | Very low |
| CR + S&C:PR + S&C | Some concerns | Low risk | No concerns | No concerns | No concerns | No concerns | Moderate |
| CR + S&C:VD + S&C | Some concerns | Low risk | No concerns | Major concerns | No concerns | No concerns | Very low |
| PR + S&C:VD + S&C | Some concerns | Low risk | No concerns | Major concerns | No concerns | No concerns | Very low |

**Table S8.3:** CINeMA Results of sprinting speed

| Comparison | Within-study bias | Reporting bias | Indirectness | Imprecision | Heterogeneity | Incoherence | Confidence rating |
| --- | --- | --- | --- | --- | --- | --- | --- |
| BA + S&C:CR + S&C | Some concerns | Low risk | No concerns | Major concerns | No concerns | No concerns | Very low |
| BA + S&C:PL + S&C | Some concerns | Low risk | No concerns | Major concerns | No concerns | No concerns | Very low |
| CR + S&C:PL + S&C | Some concerns | Low risk | No concerns | No concerns | No concerns | No concerns | Moderate |
| PL + S&C:VD + S&C | Some concerns | Low risk | No concerns | Major concerns | No concerns | No concerns | Very low |
| BA + S&C:VD + S&C | Some concerns | Low risk | No concerns | No concerns | Major concerns | No concerns | Very low |
| CR + S&C:VD + S&C | Some concerns | Low risk | No concerns | No concerns | No concerns | No concerns | Moderate |

**Table S8.4:** CINeMA Results of muscle mass

| Comparison | Within-study bias | Reporting bias | Indirectness | Imprecision | Heterogeneity | Incoherence | Confidence rating |
| --- | --- | --- | --- | --- | --- | --- | --- |
| CR + S&C:PL + S&C | Some concerns | Low risk | No concerns | Major concerns | No concerns | Major concerns | Very low |
| HMB + S&C:PL + S&C | Some concerns | Low risk | No concerns | Major concerns | No concerns | Major concerns | Very low |
| PL + S&C:PR + S&C | Some concerns | Low risk | No concerns | Major concerns | No concerns | Major concerns | Very low |
| CR + S&C:HMB + S&C | Some concerns | Low risk | No concerns | Major concerns | No concerns | Major concerns | Very low |
| CR + S&C:PR + S&C | Some concerns | Low risk | No concerns | Major concerns | No concerns | Major concerns | Very low |
| HMB + S&C:PR + S&C | Some concerns | Low risk | No concerns | Major concerns | No concerns | Major concerns | Very low |

# Appendix 9: Funnel plots

**Figure S9.1:** Funnel plot of muscle strength


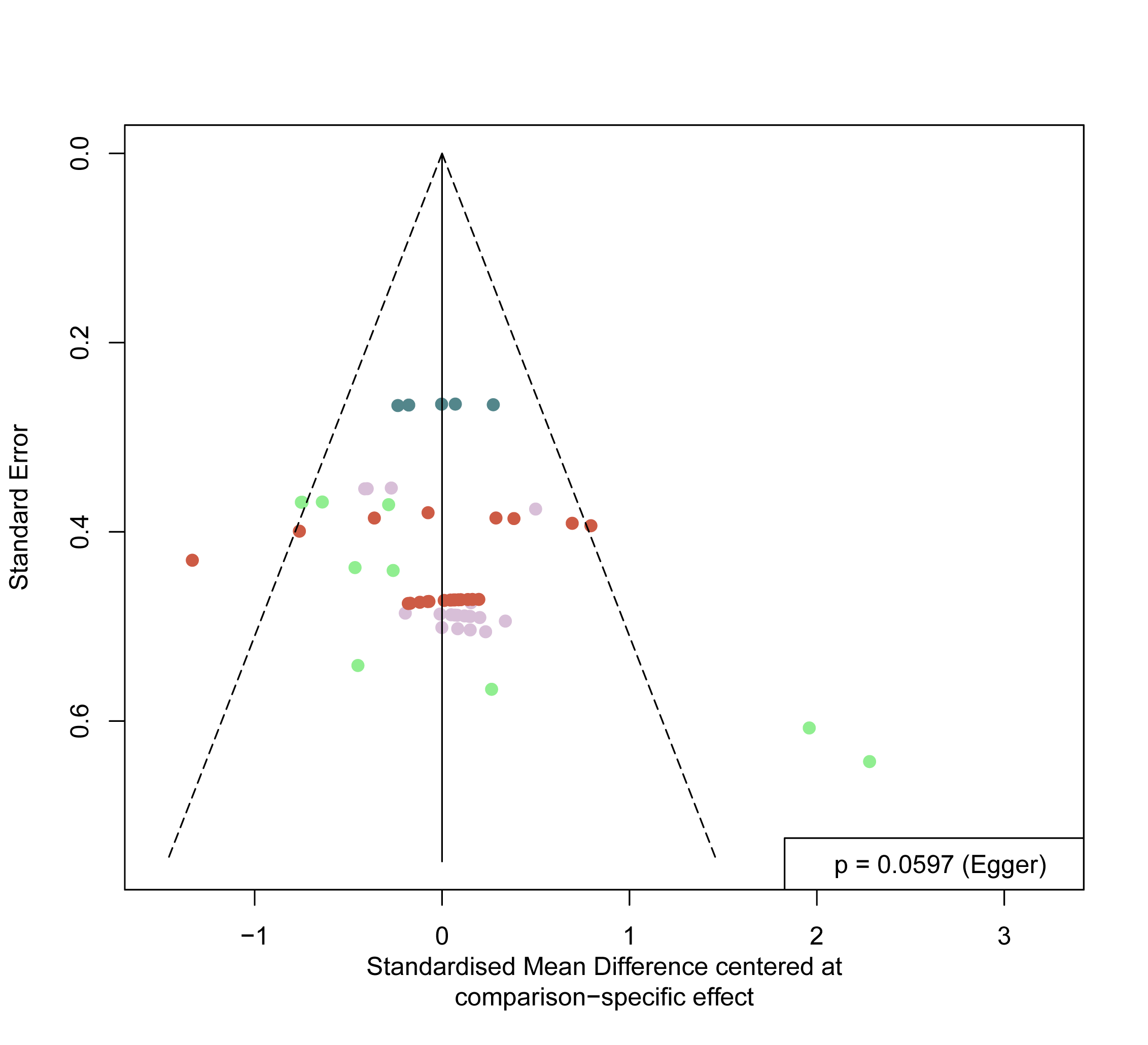


**Figure S9.2:** Funnel plot of jumping performance


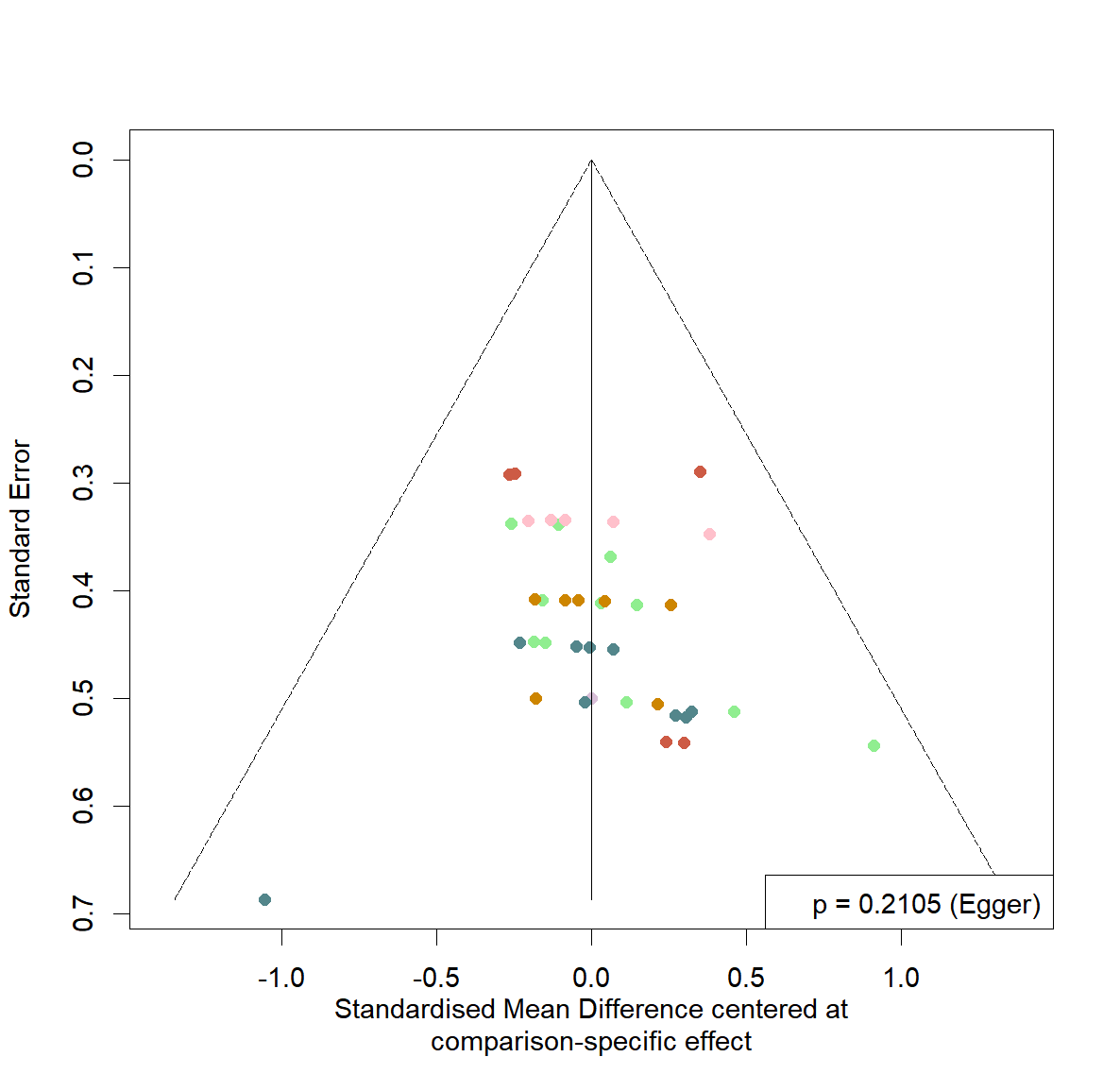


**Figure S9.3:** Funnel plot of sprinting speed





**Figure S9.4:** Funnel plot of muscle massc


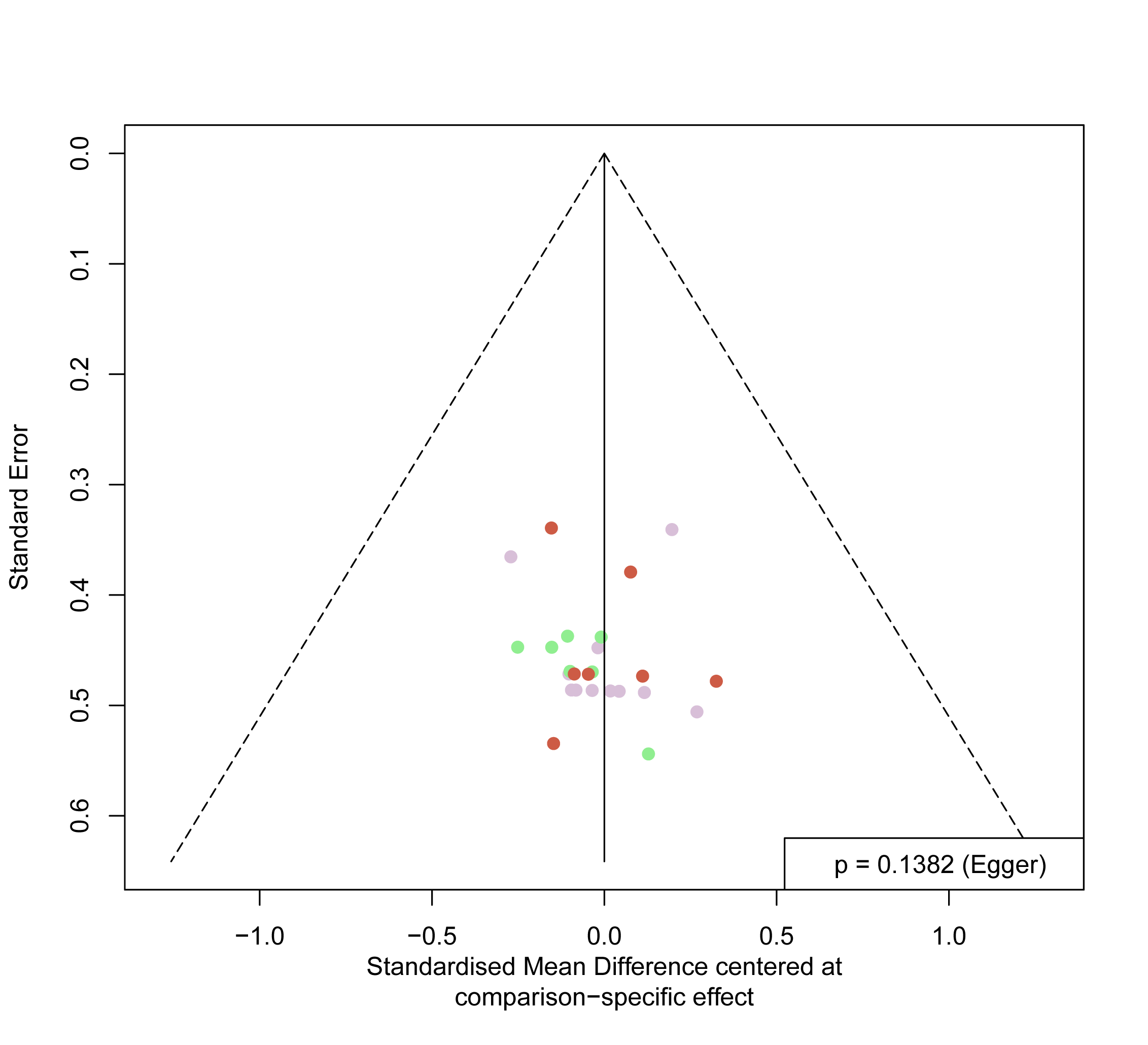


#

# Appendix S1 : Detailed CINeMA Assessment Protocol

The certainty of evidence for each network estimate derived from the network meta-analysis was graded using the Confidence in Network Meta-Analysis (CINeMA) framework [Nikolakopoulou et al., 2020, PLoS Med]. This approach systematically evaluates six distinct domains for each pairwise comparison. The overall certainty starts at 'high' and can be downgraded for concerns within each domain. The following outlines the specific criteria and decision rules applied in our assessment:

**1. Within-Study Bias**

- **Assessment Basis:** This domain considers the risk of bias from individual studies contributing to a specific network estimate. Judgments were primarily informed by the overall RoB 2.0 assessments (Low risk, Some concerns, High risk of bias – see Appendix 3) for the studies providing direct and indirect evidence for each pairwise comparison. The percentage contribution of each study to the NMA estimate, as derived from the contribution matrix (calculated using R package, was considered to weigh the impact of biased studies.
- **Rating of Concerns & Downgrading Rules:**
  - **No concerns:** If the vast majority (e.g., >75%) of the contribution to an estimate came from studies assessed as 'Low risk of bias', and minimal contribution (e.g., <10%) from studies at 'High risk of bias'.
  - **Some concerns (downgrade overall certainty by 1 level):** If a notable proportion (e.g., 25-50%) of contributing evidence came from studies with 'Some concerns', OR if a smaller but non-negligible proportion (e.g., 10-25%) came from studies at 'High risk of bias', OR if the most influential studies had 'Some concerns'.
  - **Major concerns (downgrade overall certainty by 1 or 2 levels):** If a substantial proportion (e.g., >50%) of contributing evidence came from studies with 'Some concerns', OR if a significant proportion (e.g., ≥25%) of contributing evidence came from studies at 'High risk of bias', OR if the most influential studies were at 'High risk of bias'.

**2. Reporting Bias (Publication Bias)**

- **Assessment Basis:** This domain considers the likelihood that an effect estimate is biased due to the selective publication of studies (e.g., studies with statistically significant or positive findings being more likely to be published).
  - For direct comparisons involving a sufficient number of studies (typically ≥10 studies), funnel plot asymmetry was visually inspected.
  - Egger’s regression test for small-study effects was planned for direct comparisons with ≥10 studies, with a p-value < 0.10 indicating potential bias.
  - For the overall network estimate, if key contributing direct comparisons showed evidence of reporting bias, this was considered. The impact on indirect evidence was also qualitatively assessed.
- **Rating of Concerns & Downgrading Rules:**
  - **No concerns:** Funnel plots for key contributing comparisons appeared symmetrical, and Egger’s test (if applicable) was not statistically significant.
  - **Some concerns (downgrade overall certainty by 1 level):** Slight or suspected asymmetry in funnel plots for important comparisons, or a borderline significant Egger’s test, or if there was a known high risk of publication bias in the specific field for certain types of interventions/outcomes.
  - **Major concerns (downgrade overall certainty by 1 level):** Clear and substantial asymmetry in funnel plots affecting key direct comparisons, or a statistically significant Egger’s test suggesting likely publication bias that would meaningfully alter the network estimate.

**3. Indirectness (Applicability)**

- **Assessment Basis:** This domain assesses the extent to which the evidence from included studies directly applies to the review question, considering differences in PICO (Populations, Interventions, Comparators, Outcomes) characteristics.
  - **Populations:** Differences in athlete level (e.g., elite vs. recreational), training status, sex, age, or specific sport that might modify the effect.
  - **Interventions:** Differences in supplement type (e.g., specific protein source if a general "protein" node is used), dosage, duration, or co-interventions not aligned with the review's scope.
  - **Comparators:** Differences in placebo type or control conditions that might affect relative estimates.
  - **Outcomes:** Differences in how outcomes were measured or defined that could limit direct applicability.
  - The transitivity assumption was qualitatively assessed by comparing the distribution of these potential effect modifiers across different direct comparisons forming indirect evidence. The contribution matrix helped identify influential studies for specific indirect comparisons.
- **Rating of Concerns & Downgrading Rules:**
  - **No concerns:** Included studies directly matched the PICO of the review question, and the transitivity assumption was considered plausible across relevant comparisons.
  - **Some concerns (downgrade overall certainty by 1 level):** Minor differences in PICO characteristics or some uncertainty about transitivity, but these were not judged to substantially alter the direction or magnitude of the effect. For example, if some studies included slightly different populations but the effect was expected to be similar.
  - **Major concerns (downgrade overall certainty by 1 or 2 levels):** Substantial differences in PICO characteristics that would likely and importantly alter the effect estimate (e.g., interventions applied to a very different population for which effects are known to differ significantly), or strong reasons to doubt the transitivity assumption for a key indirect comparison.

**4. Imprecision**

- **Assessment Basis:** This domain considers the uncertainty around the effect estimate, primarily judged by the width and position of the 95% confidence interval (CI) for each network pairwise comparison.
  - As established minimally clinical important differences (MCIDs) were not available for all outcome measures in the specific athletic populations included in this review, an effect estimate was generally judged as imprecise if its 95% CI was wide or crossed the line of no effect (e.g., Standardized Mean Difference (SMD) = 0).
  - The interpretation considered whether the CI included values that would lead to different clinical or practical decisions (e.g., encompassing both a meaningful benefit and no meaningful effect, or even harm).
- **Rating of Concerns & Downgrading Rules (examples):**
  - **No concerns:** The 95% CI was narrow, did not cross the line of no effect, and the point estimate suggested a clinically or practically relevant effect (or a precise null effect if that was the expectation).
  - **Some concerns (downgrade overall certainty by 1 level):** The 95% CI crossed the line of no effect but the majority of the interval and the point estimate suggested a benefit (or harm); OR the CI was wide but still predominantly on one side of no effect; OR the sample size contributing to the estimate was relatively small leading to some uncertainty.
  - **Major concerns (downgrade overall certainty by 1 or 2 levels):** The 95% CI was very wide, including both clinically/practically important benefit and important harm (or substantial benefit and substantial lack of benefit if the line of no effect is within this wide range); OR the CI was centered around the line of no effect with relatively equal portions on either side suggesting high uncertainty about the presence of any effect; OR the total number of participants or events contributing to an estimate was very small.

**5. Heterogeneity**

- **Assessment Basis:** This domain refers to the variability in the true effect sizes across studies contributing to a specific pairwise estimate, beyond that expected by chance.
  - Statistical heterogeneity was assessed using the I² statistic (e.g., thresholds of <30% as low, 30-60% as moderate, >60% as substantial or high) and the p-value from the Cochran Q test (e.g., p < 0.10 indicating significant heterogeneity) for direct comparisons and for the overall network where appropriate (e.g., τ² for between-study variance in the NMA model).
  - Clinical (differences in PICO) and methodological (differences in study design or risk of bias) diversity among studies were also qualitatively considered as potential explanations for observed statistical heterogeneity.
- **Rating of Concerns & Downgrading Rules:**
  - **No concerns:** Low statistical heterogeneity (e.g., I² < 30-40%) and no important clinical/methodological diversity suggesting inconsistency of effects.
  - **Some concerns (downgrade overall certainty by 1 level):** Moderate unexplained statistical heterogeneity (e.g., I² between 40-75%) OR important clinical/methodological diversity that likely explains some variability but uncertainty remains.
  - **Major concerns (downgrade overall certainty by 1 or 2 levels):** Substantial or high unexplained statistical heterogeneity (e.g., I² > 75%) that is not readily explained by study characteristics, suggesting true and important differences in effects across studies.

**6. Incoherence (Inconsistency)**

- **Assessment Basis:** This domain assesses the consistency between direct evidence and indirect evidence for a particular comparison within the network.
  - Evaluated primarily using node-splitting analysis, which compares the direct effect estimate with the indirect effect estimate derived from the rest of the network for comparisons forming a closed loop. A statistically significant difference (e.g., p < 0.10 from the z-test in node-splitting) was considered indicative of incoherence.
  - Global inconsistency across the network was also considered using appropriate statistical tests (e.g., design-by-treatment interaction model or the Q statistic for inconsistency from the NMA model, if applicable and interpretable).
  - For star-shaped networks or comparisons without closed loops, formal assessment of incoherence via node-splitting was not possible for those specific parts of the network.
- **Rating of Concerns & Downgrading Rules:**
  - **No concerns:** No statistically significant difference between direct and indirect evidence in relevant loops, and no other indications of important incoherence.
  - **Some concerns (downgrade overall certainty by 1 level):** Borderline statistical significance for incoherence (e.g., 0.05 ≤ p < 0.10 for node-split), or some unexplained discrepancies that raise minor doubts about the consistency of evidence.
  - **Major concerns (downgrade overall certainty by 1 level):** Statistically significant (e.g., p < 0.05 for node-split) and important incoherence between direct and indirect evidence for a key comparison, suggesting that direct and indirect evidence should not be combined or that results are highly unreliable.

**Overall Certainty of Evidence Rating:**

The overall certainty for each pairwise network estimate started at 'High'. It was then downgraded by:

- One level for each domain rated with 'Some concerns'.
- Up to two levels for each domain rated with 'Major concerns' (e.g., one level if major concerns were due to one critical flaw, two levels if concerns were very serious or pervasive across multiple aspects of that domain).
  The final certainty rating was thus 'Moderate', 'Low', or 'Very Low'. All downgrading decisions and their rationales were documented by the reviewers.
